# Randomized adaptive assessment of post COVID syndrome treatments (RAPID): a study protocol for a multicenter, randomized, controlled adaptive platform trial of treatment options for Post Covid Syndrome (PCS) on patients physical function including the first intervention specific appendix RAPID_REVIVE (reducing inflammatory activity in patients with PCS)

**DOI:** 10.1186/s13063-025-09008-0

**Published:** 2025-08-19

**Authors:** Lisa Weipert, Ralph G. Telgmann, Gabriele Anton, Thomas Asendorf, Irina Chaplinskaja-Sobol, Sandra Ciesek, Oliver A. Cornely, Sonja Drescher, Carsten Finke, Tim Friede, Julia Groth, Sabine Hanß, Wolfgang Hoffmann, Cynthia Huber, Thomas Illig, Monika Kraus, Dagmar Krefting, Sebastian Kuhn, Andreas Muehler, Matthias Nauck, Jens Schaller, Ann-Cathrin Schmidt, Georg Schmidt, Birgit Sawitzki, Ralf Tostmann, Heike Valentin, Maria Vehreschild

**Affiliations:** 1 Department II of Internal Medicine, Infectious Diseases, Goethe University Frankfurt, University Hospital Frankfurt, Frankfurt am Main, Germany; 2 Clinical Trials Unit, University Medical Center Göttingen, Göttingen, Germany; 3 Medical School OWL, Bielefeld University, Bielefeld, Germany; 4 Department of Medical Statistics, University Medical Center Göttingen, Göttingen, Germany; 5 University Medical Center Göttingen, Department of Medical Informatics, Göttingen, Germany; 6 Institute for Medical Virology, University Hospital Frankfurt, Goethe University Frankfurt, Frankfurt, Germany; 7 University of Cologne, Faculty of Medicine, University Hospital Cologne, Institute of Translational Research, Cologne Excellence Cluster on Cellular Stress Responses in Aging-Associated Diseases (CECA), Cologne, Germany; 8 Department of Neurology, Charité, University Medicine Berlin, Berlin, Germany, Berlin School of Mind and Brain, Humboldt-Universität zu Berlin, Berlin, Germany; 9 Institute of Community Medicine, Department of Healthcare Epidemiology and Community Health, University Medicine Greifswald, Greifswald, Germany; 10 Hannover Medical School, Hannover Unified Biobank, Hannover, Germany; 11 Helmholtz Centre Munich, Institute of Epidemiology, Department of Molecular Epidemiology, Munich, Germany; 12 Institute for Digital Medicine, University Hospital Giessen-Marburg, Philipps University Marburg, Marburg, Germany; 13 Immunic AG, Lochhamer Schlag 21, Gräfelfing, 82166 Germany; 14 Institute for Clinical Chemistry and Laboratory Medicine, University Medicine Greifswald, Greifswald, Germany; 15 Institute for Cardiovascular Computer-Assisted Medicine, Charité, University Medicine Berlin, Berlin, Germany; 16 Klinikum rechts der Isar, Technical University of Munich, Munich, Germany; 17 Institute of Medical Immunology, Charité, University Medicine Berlin, Berlin, Germany; 18 University of Cologne, Faculty of Medicine, University Hospital Cologne, Department I of Internal Medicine, Center for Integrated Oncology Aachen Bonn Cologne Duesseldorf (CIO ABCD) and Excellence Center for Medical Mycology (ECMM), Cologne, Germany; 19 German Centre for Infection Research (DZIF), Partner Site Bonn-Cologne, Cologne, Germany; 20 University of Cologne, Faculty of Medicine, University Hospital Cologne, Clinical Trials Centre Cologne (ZKS Köln), Cologne, Germany

**Keywords:** Post COVID syndrome, Adaptive Platform Study, Vidofludimus calcium, Physical function, Fatigue, Cognitive function

## Abstract

**Background:**

The majority of patients recovers from severe acute respiratory syndrome coronavirus type 2 (SARS-CoV-2) coronavirus disease 2019 (COVID-19) without obvious sequelae, but a significant proportion suffers long-term consequences which have been termed post COVID syndrome (PCS). Despite a wide range of considerations on treatment options in PCS and a significant number of trials initiated, only very few results from randomized controlled trials are currently available. In conclusion, there is an evident medical need to identify treatments for patients with PCS.

**Methods:**

The primary objective of the platform trial RAPID is to assess the impact of different PCS treatments on the overall physical function of patients. Designed as a master protocol, RAPID contains all information that is generic to this adaptive platform trial. Current and future study treatments are specified in intervention-specific appendices (ISA). The first ISA, RAPID_REVIVE is presented in this manuscript. General sections of the master protocol are named as such.

RAPID_REVIVE is a double-blind, placebo-controlled, phase II clinical trial evaluating antiviral PCS treatment with vidofludimus calcium (IMU-838). Patients are randomized at a 1:1 ratio to 45 mg/day vidofludimus calcium (22.5 mg for the first 7 days) or placebo during an initialization phase and thereafter using a response-adaptive randomization procedure. The trial includes a screening period of 7 days, a double-blind treatment period of 56 days and a follow-up period of 28 days. The primary outcome is the intra-patient change in physical function measured by the Short Form-36 Physical Function (SF-36-PF) from baseline to day 56. Secondary endpoints include mental and physical health, intensity of fatigue, severity of mental disorder symptoms, and cognitive function.

**Discussion:**

PCS is a major problem for global health care and the identification of treatment options is urgently needed. Currently, PCS patients are in a situation without evidence-based treatment options, and quality of life, and often mental health are significantly impaired. The purpose of RAPID is to establish an adaptive platform trial protocol which will concert and quicken clinical trials to evaluate the efficacy and safety of different potential treatments for PCS with the aim to expand the very limited evidence base for the treatment of PCS.

**Trial registration:**

EU Clinical Trials Register (CTIS) ID: 2024–511628-16–00 (RAPID_REVIVE). Registered on 18.03.2024.

**Supplementary Information:**

The online version contains supplementary material available at 10.1186/s13063-025-09008-0.

## Administrative information

Note: the numbers in curly brackets in this protocol refer to SPIRIT checklist item numbers. The order of the items has been modified to group similar items (see http://www.equator-network.org/reporting-guidelines/spirit-2013-statement-defining-standard-protocol-items-for-clinical-trials/).

**Table:** 

Title {1}	Randomized adaptive Assessment of Post COVID syndrome treatments (RAPID), A Multicenter, Randomized, Controlled Adaptive Platform Study on the Safety and Efficacy of Investigational Therapeutics and Interventions for the treatment of Post Covid Syndrome (PCS) with one intervention specific subprotocol RAPID_REVIVE
Trial registration {2a and 2b}.	EU Clinical Trials Register (CTIS) ID: 2024–511628-16–00 (RAPID_REVIVE), date of registration: 18–03-24
Protocol version {3}	V 2.0, 13.05.2024
Funding {4}	This publication was funded by the German Federal Ministry of Education and Research (BMBF) Network of University Medicine 2.0: “NUM 2.0", Grant No. 01KX2121, Projects: NAPKON-TIP and NAPKON-TIP: RAPID trial
Author details {5a}	Lisa Weipert^1^, Ralph G. Telgmann^2^, Gabriele Anton^3^, Thomas Asendorf^4^, Irina Chaplinskaja-Sobol^5^, Sandra Ciesek^6^, Oliver A. Cornely^7^, Sonja Drescher^4^, Carsten Finke^8^, Tim Friede^4^, Julia Groth^11^, Sabine Hanß^5^, Wolfgang Hoffmann^9^, Cynthia Huber^4^, Thomas Illig^10^, Monika Kraus^11^, Dagmar Krefting^5^, Sebastian Kuhn^12^, Andreas Muehler^13^, Matthias Nauck^14^, Jens Schaller^15^, Ann-Cathrin Schmidt^1^, Georg Schmidt^16^, Birgit Sawitzki^17^, Ralf Tostmann^2^, Heike Valentin^9^, Maria Vehreschild^1^ ^1^Department of Internal Medicine, Infectious Diseases, University Hospital Frankfurt, Goethe University Frankfurt, Frankfurt am Main, Germany ^2^Clinical Trials Unit, University Medical Center Göttingen, Göttingen, Germany ^3^Medical School OWL, Bielefeld University, Bielefeld, Germany ^4^Department of Medical Statistics, University Medical Center Göttingen, Göttingen, Germany ^5^University Medical Center Göttingen, Department of Medical Informatics, Göttingen, Germany ^6^Institute for Medical Virology, University Hospital Frankfurt, Goethe University Frankfurt, Frankfurt, Germany ^7^University of Cologne, Faculty of Medicine, University Hospital Cologne, Institute of Translational Research, Cologne Excellence Cluster on Cellular Stress Responses in Aging-Associated Diseases (CECAD), Cologne, Germany University of Cologne, Faculty of Medicine, University Hospital Cologne, Department I of Internal Medicine, Center for Integrated Oncology Aachen Bonn Cologne Duesseldorf (CIO ABCD) and Excellence Center for Medical Mycology (ECMM), Cologne, Germany German Centre for Infection Research (DZIF), Partner Site Bonn-Cologne, Cologne, Germany University of Cologne, Faculty of Medicine, University Hospital Cologne, Clinical Trials Centre Cologne (ZKS Köln), Cologne, Germany ^8^Department of Neurology, Charité, University Medicine Berlin, Berlin, Germany, Berlin School of Mind and Brain, Humboldt-Universität zu Berlin, Berlin, Germany ^9^Institute of Community Medicine, Department of Healthcare Epidemiology and Community Health, University Medicine Greifswald, Greifswald, Germany ^10^Hannover Medical School, Hannover Unified Biobank, Hannover, Germany ^11^Helmholtz Centre Munich, Institute of Epidemiology, Department of Molecular Epidemiology, Munich, Germany ^12^Institute for Digital Medicine, University Hospital Giessen-Marburg, Philipps University Marburg, Marburg, Germany. ^13^Immunic AG, Lochhamer Schlag 21, 82,166 Gräfelfing, Germany ^14^Institute for Clinical Chemistry and Laboratory Medicine, University Medicine Greifswald, Greifswald, Germany ^15^Institute for Cardiovascular Computer-Assisted Medicine, Charité, University Medicine Berlin, Berlin, Germany ^16^Klinikum rechts der Isar, Technical University of Munich, Munich, Germany ^17^Institute of Medical Immunology, Charité, University Medicine Berlin, Berlin, Germany
Name and contact information for the trial sponsor {5b}	Goethe University Frankfurt represented by Prof. Dr. med. Maria J.G.T. Vehreschild University Hospital Frankfurt Innere Medizin II, Infektiologie Theodor-Stern-Kai 7 60,590 Frankfurt, Germany.
Role of sponsor {5c}	The funder BMBF did not influence the study design and will not influence the collection, management, analysis, and interpretation of data; writing of the report; and the decision to submit the report for publication.

## Introduction

### Background and rationale {6a}

#### Post COVID syndrome

The majority of patients recover from severe acute respiratory syndrome coronavirus type 2 (SARS-CoV-2) coronavirus disease 2019 (COVID-19) without obvious sequelae, but a significant proportion suffers long-term consequences which have been termed post COVID syndrome (PCS) [1–5]. About 10–20% of patients who experienced COVID-19 report persistent or new symptoms, according to the World Health Organization (WHO), which is a major problem for global health care [6, 7]. The diverse clinical symptoms may be explained by the broad expression of the angiotensin converting enzyme 2 receptor (ACE2). This receptor is required by SARS-CoV-2 to enter the cell and is ubiquitously expressed in many tissues (lung, kidney, small intestine, olfactory neuroepithelium, heart, testis, “substantia nigra,” and muscle cells) [8, 9]. The presence of ACE2 receptors in the vascular endothelium and the gut, as well as the occurrence of accompanying inflammatory and immunological processes provide a hypothesis potentially explaining the high diversity of clinical manifestations of COVID-19. These include fatigue, muscle pain, dyspnea, headache, olfactory/gustatory dysfunction, cognitive dysfunction (attentional and executive impairments), depression, and anxiety [5, 10–12]. In a significant number of cases, the above-described signs and symptoms persist over longperiods and potentially life-long.

#### Rationale for adaptive platform trial

Despite a wide range of considerations on treatment options in PCS and a significant number of trials initiated, only very few results from randomized controlled trials are currently available. At the same time, numerous hypotheses concerning potentially effective treatment strategies are slowly emerging from ongoing research [13]. This adaptive platform study evaluates the efficacy and safety of several different potential treatments which are being developed for post COVID syndrome (PCS). The platform study design enables multiple study interventions to be evaluated in a clinical study in a simultaneous manner. Platform study designs have specific operating characteristics which need to be carefully considered and balanced against the complexity which is introduced. For this study, the design is anticipated to provide a more efficient means of evaluating novel therapies for the treatment of PCS. The first intervention-specific appendix (ISA) RAPID_REVIVE (Reducing Inflammatory Activity in Patients with PCS syndrome) of the overarching master protocol RAPID (Randomized adaptive Assessment of Post COVID syndrome treatments) is presented here in this manuscript. Generic sections of the master protocol are named as such.

#### Rationale of RAPID_REVIVE

An increased level of inflammatory activity has been shown to play a key role in the pathogenesis and chronic clinical picture of PCS. There are, however, multiple factors that have been hypothesized to trigger aberrant inflammatory activation [14]. These include, among others, two potentially relevant causes of PCS and thus suitable treatment targets, persistence of SARS-CoV-2 for > 6 months after COVID-19, as well as reactivation of Epstein-Barr virus (EBV) and other dormant viruses [14, 15]. In detail, persistent “spike 1” (S1) protein could be found in CD16 + monocytes from PCS patients [16], and the intestine has been shown to harbor SARS-CoV-2 long after a COVID-19 infection [17, 18]. Furthermore, patients receiving antiviral treatment for their acute COVID-19 disease have been shown to experience PCS less frequently and/or severely than untreated patients [19–21]. In a case series, in which individuals were treated with Nirmatrelvir/Ritonavir at various stages following infection, improvement of PCS symptoms was shown [22]. This argues for the SARS-CoV-2 reservoir being an important determinant in causing persistent inflammation as a pathogenetic factor in PCS. Besides persistence of SARS-CoV-2 itself, the reactivation of other latent viral infections, e.g., EBV may be associated with PCS. In line with this hypothesis, there is an increasing number of studies reporting virus reactivation including EBV in patients with PCS. Depending on the study, EBV reactivation was observed in 50–95% of patients, indicated by anti-EBV-antibodies or EBV viremia [1, 23–26]. Virus reactivation may occur as early as 2 weeks of SARS-CoV-2 infection [1]. EBV reactivation was also associated with fatigue in hospitalized and non-hospitalized patients [23]. Besides viral persistence and reactivation, other factors may also contribute to the onset of PCS. Initial exposure to SARS-CoV-2 may set off a cascade of autoimmune responses. Such initial inflammatory activation may be reinforced through the presence of a disbalanced gut microbiota or a procoagulatory response of the coagulation cascade [14]. Concerning the need for identification of treatment targets for PCS, RAPID_REVIVE focuses on addressing the pathomechanisms associated with viral persistence and reactivation to improve clinical signs and symptoms of PCS.

#### IMU-838

Vidofludimus calcium (IMU-838) is a small molecule that selectively inhibits the enzyme dihydroorotate dehydrogenase (DHODH), which catalyzes the rate-limiting step of the de novo pyrimidine synthesis. It represents a novel chemical class with no structural similarity to other known, commercially available DHODH inhibitors (i.e., leflunomide, teriflunomide) [27]. IMU-838 specifically affects pyrimidine synthesis in cells with exceptionally high need for nucleotides, while other host cells can cover their requirement for pyrimidines via salvage pathways. Cell types in high need of pyrimidine include cancer and virus-infected cells, but also hyperactive cells in chronic inflammatory diseases [28]. In the latter, inhibiting DHODH causes secondary metabolic stress that leads to a reduction of pro-inflammatory cytokine release. Moreover, activation of metabolic stress pathways results in the induction of an IFN-independent innate immune response. Thereby, IMU-838 prevents viral replication directly by diminishing the cellular pyrimidine pool and indirectly by an IFN-independent upregulation of IFN-stimulated genes with antiviral activity [28]. Of note, the antiviral effect of DHODH inhibition is independent of virus-specific proteins and their structure. Thus, IMU-838 exhibits a broad-spectrum antiviral activity against different viruses (including SARS-CoV-2 variants, EBV, hepatitis C virus, human immunodeficiency virus (HIV-1), human cytomegalovirus, and influenza A virus) with an antiviral efficacy in the low micromolar range in vitro [28]. IMU-838 was selected for this trial based on existing clinical and safety data in COVID-19 patients, as well as its broad antiviral spectrum. In a phase 2 trial of IMU-838, 223 hospitalized COVID-19 patients were randomized to standard-of-care plus either placebo (*n* = 112) or IMU-838 45 mg (*n* = 111). Data received from this trial indicate a shorter time to clinical improvement after treatment with IMU-838 and a correlation between IMU-838 trough levels and the number of days to clinical recovery [20]. Furthermore, results from a post hoc analysis of PCS symptoms indicated a potential contribution of IMU-838 to the prevention of long-term fatigue, one of the most common post COVID-19 symptoms. At initial clinical and viral remission from the SARS-CoV-2 infection, 80% patients who received placebo reported fatigue compared to 50% patients receiving IMU-838 45 mg. Fatigue decreased in both treatment groups in the following 9–17 weeks to 33% in placebo and 17% in IMU-838 recipients. In the same trial, IMU-838 treatment in COVID-19 patients was found to be well tolerated [20]. Premature study discontinuations for any reason or related to study treatment were similar between IMU-838 and placebo. Likewise, rates of treatment emergent adverse events (AEs) of grade 3 or higher and serious adverse events (SAEs) were low and comparable between IMU-838 and placebo. In conclusion, IMU-838 potentially acts on three potential underlying causes of PCS, namely persistence of a SARS- CoV-2 reservoir, reactivation of latent viral infections, and chronic upregulation of inflammation. Concerning the approach of using antiviral treatments to treat PCS, several trials with an intention to assess the use of 10 days nirmatrelvir have been registered (NCT05595369, NCT05576662, NCT05823896, NCT05852873), but no trial assessing the efficacy of IMU-838 in this indication has been set up so far, despite its multiple potential mechanisms of action against PCS. The first ISA of the RAPID adaptive platform trial (APT) will fill this gap. IMU-838 does not only display activity against SARS-CoV-2, but also against other viruses (e.g., EBV, cytomegalovirus (CMV)) that may reactivate in the context of COVID 19 and thus initiate PCS [14].

### Objectives {7}

The primary generic objective of the RAPID platform trial is to compare the impact of the individual PCS treatment to control on patient overall physical function as measured by the Short Form-36 Physical Function (SF-36-PF) from baseline to day 56 in adults with PCS. While this primary objective also applies to each ISA, secondary objectives are specified for each ISA.

Secondary objectives of the ISA RAPID_REVIVE that is presented in this manuscript are to compare the impact of PCS treatment with IMU-838 to control on patient overall mental and physical health at days 28, 56, and 84, to compare the impact of PCS treatment with IMU-838 to control on patient- and physician-reported outcomes measuring key PCS neuropsychiatric symptoms, on patient- and physician-reported outcomes measuring key physical PCS symptoms and on autonomic function and physical activity parameters. With the objective to identify and preselect patients that might benefit from the selected intervention, blood, fecal, and imaging biomarkers will be evaluated as exploratory endpoints. The safety objective is to evaluate safety and tolerability of IMU-838 (see Table 2).

## Methods: participants, interventions and outcomes

### Trial design {8}

RAPID is designed as an open end APT with the option of adding and removing interventional arms. The master protocol contains generic information like, i.e., eligibility criteria, randomization rules, endpoints, and the overarching statistical approach. The specific trial domains of care and their interventions are defined in their corresponding appendices.

**Fig. 1:**
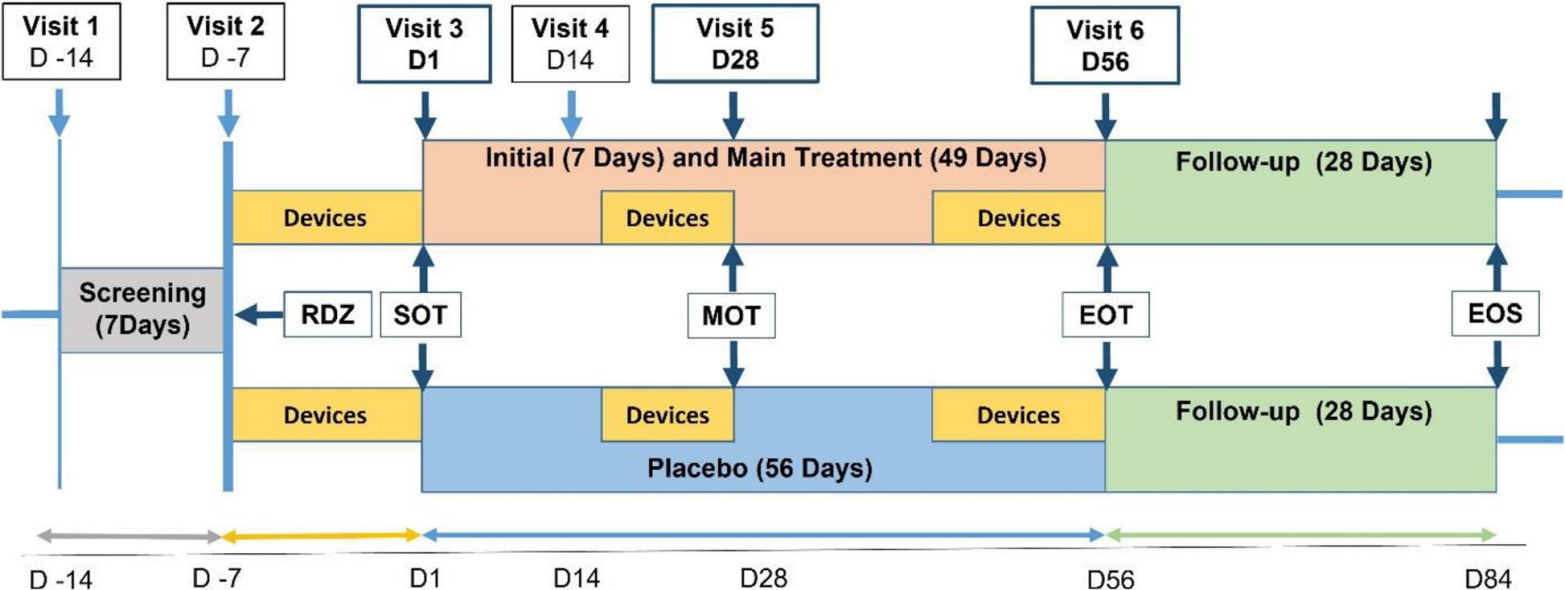
Trial flowchart of RAPID_REVIVE from Screening to follow up. RDZ = randomization; SOT = start of treatment; MOT = middle of treatment; EOT = end of treatment; EOS = end of study; Devices = Measurement of autonomic function and physical activity parameters by medical devices (see {18a}); Initial Treatment = 7-day initiation phase with 22.5 mg/day IMU-838; Main Treatment = 45-day treatment phase with 45 mg/day IMU-838

RAPID_REVIVE is an intervention-specific appendix (ISA) linked to the master protocol RAPID targeting treatment of PCS. RAPID_REVIVE is a phase 2 randomized, multi-center, placebo-controlled, double-blind, parallel group, superiority clinical trial with two arms. Patients are randomized 1:1 to receive either IMU-838 45 mg/day (22.5 mg/day initiation dose) or placebo for 56 days in a double-blind fashion during an initialization phase and thereafter using a response-adaptive randomization procedure. They are followed up until day 84. A flowchart of the trial is presented in Fig. 1.

### Study setting {9}

Patients are recruited from 11 hospitals across Germany. If necessary, additional qualified sites can be included during trial conduct. All trial sites participated in patient recruitment for the 3 NAPKON (Nationales Pandemie Kohorten Netz) cohorts and benefit from the already established, central trial infrastructures. The aim of NAPKON was to recruit a high-quality cohort of patients with coronavirus disease 2019 (COVID-19) for in-depth phenotyping of the disease. NAPKON recruited all patient types and at all healthcare facilities in Germany as part of the three cohorts SÜP (Sektorenübergreifende Plattform), HAP (Hochauflösende Plattform), and POP (Populationsbasierte Plattform). The project also included the creation of infrastructures for the collection, management and provision of data, image data, and biosamples. A current list of study sites is published online on the RAPID_REVIVE website (https://www.netzwerk-universitaetsmedizin.de/projekte/napkon-tip/studie-rapid-revive).

### Eligibility criteria {10}

#### Inclusion criteria

Patients eligible for inclusion in RAPID_REVIVE must meet *all* of the following criteria (criteria 1, 2, 5, and 6 are generic to RAPID):

1. Male or female patients ≥ 18 years of age
2. Symptoms consistent with PCS that began within 4 weeks of the index infection and persisted for > 12 weeks. The identified symptoms of PCS cannot be attributed to other intervening diagnoses or medications and did not exist prior to the acute COVID preceding PCS.
3. Moderate to severe overall disability, defined as a Bell Scale [29] of 20–60
4.  ≥ 2 of the following post-COVID symptoms, defined as:
  - Fatigue, defined as an FSS score ≥ 36
  - Cognitive impairment, defined as a MoCA score between 10 and 25
  - Shortness of breath, defined as a mMRC ≥ 2
  - Orthostatic/autonomic dysfunction, defined as the following results in the PST:
    i. A sustained heart rate increase of ≥ 30 bpm within 10 min. of standing or and/or a heart rate reaching > 120 bpm within 10 min. of standing *and*
    ii. Absence of a sustained 20 mmHg decrease in systolic blood pressure within 10 min of standing
5. Written informed consent obtained according to international guidelines and local laws
6. Ability to understand the nature of the trial and the trial-related procedures and to comply with them
7. Ability to provide and use a smartphone, tablet, or other device for download and installation of the medical device software used in the trial
8. Willingness to not connect medical devices used in this trial to any other app that is not defined in this protocol, especially not to Garmin Connect
9. Willingness to abstain from changes in the type, dosage, and frequency of concomitant medications through day 84
10. Use of a highly effective method of contraception correctly and consistently, as applicable, during trial treatment and for 28 days after the final dose (applicable to individuals of childbearing potential and participating men whose partners may become pregnant)
11. Female patients of childbearing potential, must have a negative pregnancy test (at Screening-V1 (blood test; see Table 1) and before the first Investigational Medicinal Product (IMP) intake (day 1 blood or urine test) AND agreement of not to attempt to become pregnant AND agreement of not to donate ova AND usage of highly effective forms of birth control (as defined by Recommendations related to contraception and pregnancy testing in clinical trials- Heads of Medicines Agencies (HMA) [30]).
12. Male patients must agree not to father a child or to donate sperm starting at Screening-V1, throughout the clinical study, and for 30 days after the last intake of the IMP. Male patients must also:
  - Abstain from sexual intercourse with a female partner (acceptable only if it is the patient’s usual form of birth control/lifestyle choice), or
  - Use adequate barrier contraception during treatment with the IMP and until at least 30 days after the last intake of the IMP, and
  - If they have a female partner of childbearing potential, the partner should use a highly effective contraceptive method as outlined above
  - If they have a pregnant partner, they must use condoms while taking the IMP to avoid exposure of the fetus to the IMP.

**Table 1:** Restricted medications recommended based on previous studies on IMU-838 interaction potential with drug-metabolizing enzymes and transporters

Class or type	Medication/patient population	Reason
CYP2C8 strong inducers	Rifampicin, barbiturates (e.g., phenobarbital), carbamazepine, ritonavir	Potentially decrease exposure to IMU-838 and may thus decrease efficacy
CYP2C8 strong inhibitors	Gemfibrozil, clopidogrel	Potentially increase exposure to IMU-838 and may thus increase the risk for AEs; however uncertain due to alternative metabolic pathways of IMU-838
BCRP substrates with a narrow therapeutic window	Rosuvastatin (maximum dose: 10 mg/day), methotrexate (maximum dose: 17.5 mg/week), mitoxantrone, sulfasalazine, topotecan, daunorubicin, doxorubicin, warfarin	IMU-838 may inhibit transport of BCRP substrates and lead to increased exposure in plasma and/or hepatocytes and thus increased risk for substrate’s side-effects
BCRP strong inducers	DMARDs such as methotrexate (maximum dose: 17.5 mg/week), sulfasalazine	Potentially decrease exposure to IMU-838 and may thus decrease efficacy
BCRP strong inhibitors	Immunosuppressants (cyclosporine A, tacrolimus, sirolimus), azole antifungals (ketoconazole, itraconazole, fluconazole), proton pump inhibitors (omeprazole, pantoprazole), NSAIDs (ibuprofen, naproxen, salicylates)	Potentially increase exposure to IMU-838 and may thus increase the risk for AEs
UGT1A1 inhibitors	Atazanavir, canagliflozin, pazopanib, regorafenib, sorafenib, tocilizumab, tranilast	Combination therapy of IMU-838 and other UGT1A1 inhibitors may lead to disturbance of the bilirubin metabolism and subsequently to hyperbilirubinemia
Other safety precautions/restrictions for patient population related to DDI potential		
UGT1A1	Patients with reduced UGT1A1 activity (e.g., Gilbert’s syndrome) to be excluded from clinical studies with IMU-838	In patients with reduced UGT1A1 activity: Risk of hyperbilirubinemia

Abbreviations: *AE* adverse events, *BCRP* breast cancer resistance protein, *CYP* cytochrome, *DDI* drug-drug interaction, *DMARDs* disease-modifying anti-rheumatic drugs, *UGT1A1* uridine-diphosphate glucuronosyltransferase 1A1

### Exclusion criteria

Patients eligible for RAPID_REVIVE must *not* meet any of the following criteria (criteria 17, 18, 19, and 21 are generic to RAPID):

1. Known or planned pregnancy; nursing period
2. Participation in any other interventional clinical trial within the last 30 days before the start of this trial
3. Simultaneous participation in other interventional trials which could interfere with this trial (simultaneous participation in registry and diagnostic trials is allowed)
4. Patient without legal capacity who is unable to understand the nature, significance, and consequences of the trial
5. Previous participation in this trial
6. Known or persistent abuse of medication, drugs, or alcohol
7. Person who is in a relationship of dependence/employment with the sponsor or the investigator
8. Persons deprived of liberty or placed in an institution by judicial or administrative order
9. Presence of the following laboratory values at screening
  Platelet count < 100,000/mm^3^ (< 100 × 10.^9^/L)
  Neutrophil count < 1500/mm^3^ (1.5 × 10.^9^/L)
  Serum creatinine > 1.5 × upper limit of normal (ULN)
  Total bilirubin, GOT, GPT, or gGPT > 1.5 × ULN
  Serum uric acid levels > 1.2 × ULN
  Indirect (unconjugated) bilirubin > 1.2 × ULN
10. Known or suspected Gilbert syndrome (Morbus Meulengracht)
11. Severe impairment of liver function (Child Pugh class C)
12. Known history of nephrolithiasis or underlying condition with a strong association of nephrolithiasis, including hereditary hyperoxaluria or hereditary hyperuricemia
13. History or clinical diagnosis of gout
14. History of malignancy of any organ system (other than localized basal cell carcinoma of the skin or adequately treated cervical cancer), treated or untreated, within the past 5 years, regardless of whether there is evidence of local recurrence or metastases.
15. History of medically significant active, chronic systemic infections (not considering the SARS-CoV-2 infection) within 6 months before day 1, including, but not limited to tuberculosis, hepatitis B, C, or D, and HIV
16. History or presence of serious or acute heart disease such as uncontrolled cardiac dysrhythmia or arrhythmia, uncontrolled angina pectoris, cardiomyopathy, or uncontrolled congestive heart failure (New York Heart Association [NYHA] class 3 or 4)
17. History or presence of any major medical or psychiatric illness which cannot be controlled by medication (e.g., severe depression, schizophrenia, psychotic disorder), history of suicide attempt, or current suicidal ideation, if any of those conditions in the opinion of the investigator could create undue risk to the patient or could affect adherence with the trial protocol
18. Ongoing SARS-CoV-2 infection or positive test for SARS-CoV-2 within 14 days prior to enrolment
19. Pre-COVID history of chronic fatigue syndrome or other fatigue syndromes that are due to associated diseases (e.g., cancer, autoimmune diseases)
20. Prior use of IMU-838 or a drug prescribed to treat COVID-19 within 30 days
21. Vaccination for COVID-19 within 28 days prior to enrollment, or other vaccines (influenza, shingles, etc.) within 14 days of enrollment, or planned use of any vaccine until day 84
22. Any concomitant disease impairing efficacy endpoint analysis, in the opinion of the investigator
23. Any use of the following concomitant medications is prohibited during screening:
  Any medication known to significantly increase urinary elimination of uric acid, in particular lesinurad, as well as uricosuric drugs such as probenecid
  Treatments for any malignancy, in particular irinotecan, paclitaxel, tretinoin, bosutinib, sorafinib, enasidenib, erlotinib, regorafenib, pazopanib, and nilotinib
  Any drug significantly restricting water diuresis, in particular vasopressin and vasopressin analogs
  Rosuvastatin at doses of > 10 mg/day
  Methotrexate at doses of > 17.5 mg/week
24. Hypersensitivity to the active substance or to any of the excipients.

### Who will take informed consent? {26a}

If a patient appears to be eligible for the trial, the investigator at the participating sites informs the patient about the trial and ask the patient for their written consent. It is imperative that written consent is obtained prior to any trial-specific procedures. Patients are enrolled in the trial after informed consent has been obtained between day − 7 and day − 14.

### Additional consent provisions for collection and use of participant data and biological specimens {26b}

RAPID_REVIVE includes an optional biosample collection as well as a subsequent use of remaining study samples and medical data collected during the study, respectively. These samples and data can be used for further research projects that have been approved in the use and access process of the Netzwerk Universitätsmedizin (NUM). Scientists that requested samples or data for their own research project through this use and access process must obtain a separate ethics vote for their project. This must be applied for at the ethics committee of their location.

If a consent for secondary use has been signed by the patient, samples obtained for biomarker assessments but not completely used in the course of the study are stored for the biobank. Additionally, one EDTA blood sample and one saliva sample will be obtained for patients with signed consent for secondary use.

The separate ICF for secondary use (Attachment 3) includes in language understandable to laypeople an outline of the important aspects in this regard such as the scientific value of secondary use, responsibilities, the technical infrastructure for data processing, and the regulatory framework including ethical and legal aspects, data protection, and the use and access process. In this context, particular attention is paid to the distinction between study and secondary use. The ICF for secondary use can be employed generically for other arms of the RAPID platform trial, provided that it is funded by the NUM.

## Interventions

### Explanation for the choice of comparators {6b}

A placebo arm is included due to regulatory recommendations to evaluate benefits and adverse effects in randomized, double-blind, placebo-controlled studies. Because additional treatment is allowed during the entire trial with few exceptions, a placebo arm is justified.

### Intervention description {11a}

Overall treatment time is 56 days. Patients in the treatment arm receive IMU-838 22.5 mg/day for a 7-day initiation phase and subsequently IMU-838 45 mg/day for the remaining treatment period. Patients in the control arm receive placebo for 56 days. IMU-838 and placebo (identical to the IMU-838 tablets in appearance, constitution of inactive ingredients, and packaging) are administered once daily as oral tablets. A single tablet is taken once daily in the morning in fasted state (no food after midnight unrestricted intake of water is always allowed) with one glass of water approximately 15 min to 1 h before breakfast.

### Criteria for discontinuing or modifying allocated interventions {11b}

Patients receive treatment from day 1 until day 56 or until discontinuation due to intolerable toxicity, withdrawal of consent, loss to follow-up, death, or termination of the trial. They are followed up until day 84 after initiation of treatment. Dose modifications are not permitted during the trial. Any deviation has to be previously discussed with the sponsor unless it concerns patient’s safety. All interruptions must be recorded on the appropriate electronic Case Report Form (eCRF) page.

### Strategies to improve adherence to interventions {11c}

Face-to-face adherence reminder sessions (patients and investigator or another appropriate individual who is designated by the investigator) take place at the initial product dispensing and each trial visit thereafter. This session includes:

- The importance of following trial guidelines for adherence to once daily intake of IMP.
- Instructions about taking IMP including timing, storage, and importance of taking IMP, and what to do in the event of a missed dose.
- Notification that there will be an IMP count at the day 56 trial visit (used/empty/unsealed/damaged and/or unused packages have to be shown to the investigator).
- Importance of calling the clinic if experiencing problems related to IMP such as symptoms or loss or damage of packages.

Subsequent sessions occur at the follow-up visits. Participants are asked about any problems they are having taking their IMP. There are brief discussion of reasons for missed doses and simple strategies for enhancing adherence, e.g., linking IMP taking to meals or other daily activities. Participants will have an opportunity to ask questions and key messages from the initial session will be reviewed as needed.

### Relevant concomitant care permitted or prohibited during the trial {11d}

#### Permitted concomitant therapy requiring caution and/or action

Restricted concomitant medications are not generally prohibited (Table 1), but use should be restricted as far as possible in terms of dose and treatment duration. In accordance with the prescribing information, the lowest effective dosage for the shortest duration should be applied based on the individual patient treatment goals whenever possible. Alternatives to these drugs should be considered and patients should be carefully monitored for any indication of overdose and/or toxicity. Care should be exercised when using medications that are substrates of the breast cancer resistance protein (BCRP) transport system, especially where the elimination of the medication depends on the BCRP transport system. Patients should be closely monitored for signs and symptoms of excessive exposure to medicinal products and the dosing of these medicinal products should be carefully considered. This is particularly true for statins, and their dose should be lowered to the lowest possible dose. Specifically, doses of rosuvastatin are not to exceed 10 mg daily.

### Prohibited concomitant therapy

Any use of the following concomitant medications is prohibited during screening and throughout the duration of the trial (exclusion criteria 23):

- Any medication known to significantly increase urinary elimination of uric acid, in particular lesinurad, as well as uricosuric drugs such as probenecid.
- Treatments for any malignancy, in particular irinotecan, paclitaxel, tretinoin, bosutinib, sorafinib, enasidenib, erlotinib, regorafenib, pazopanib, and nilotinib.
- Any drug significantly restricting water diuresis, in particular vasopressin and vasopressin analogs.
- Rosuvastatin at doses of > 10 mg/day.
- Methotrexate at doses of > 17.5 mg/week.

If such agents are required for a patient, the patient has to discontinue the trial treatment and the given prohibited therapy has to be recorded in the eCRF.

### Provisions for post-trial care {30}

After end of the trial, the therapy of PCS will be performed according to the trial site’s routine. A subject insurance according to applicable law has been taken out with for all subjects participating in the clinical trial. The insurance covers all harms that resulted from trial participation.

### Outcomes {12}

Table 2 shows the objectives and related endpoints.

**Table 2:** Objectives and related endpoints

Objective	Endpoint
RAPID	
Primary	
Compare the impact of PCS treatment to control on patient overall physical function	Intra-patient change in physical function as measured by the Short Form-36 Physical Function (SF-36-PF)
Secondary	
Compare the impact of PCS treatment on secondary objecttives specified by ISA	Intra-patient change in parameter specified by ISA
Safety	
To evaluate safety and tolerability of treatment	Type, frequency, and severity of AEs and SAEs
RAPID_REVIVE	
Primary	
Compare the impact of PCS treatment with IMU-838 to control on patient overall physical function at day 56	Intra-patient change in physical function as measured by the Short Form-36 Physical Function (SF-36-PF) from baseline to day 56
Secondary	
Compare the impact of PCS treatment with IMU-838 to control on patient overall mental and physical health at days 28, 56, and 84	Intra-patient change in overall mental and physical health as measured by the SF-36-PF; from baseline to days 28, 56, and 84
Compare the impact of PCS treatment with IMU-838 to control on patient- and physician-reported outcomes measuring key PCS neuropsychiatric symptoms	Intra-patient change from baseline to days 28, 56, and 84 in - Intensity of fatigue and incapacitation measured by the FSS - Severity of mental disorder symptoms such as depression, anxiety, somatization, and distress measured by the PHQ modules PHQ-9, PHQ-15, and PHQ-stress and GAD-7 Intra-patient change from baseline to days 28 and 56 in - Cognitive function measured by the MoCa
Compare the impact of PCS treatment with IMU-838 to control on patient- and physician-reported outcomes measuring key physical PCS symptoms	Intra-patient change from baseline to days 28, 56, and 84 in - Severity of dyspnea, measured by the mMRC - PEM frequency, strength and severity as measured by the PEM questionnaire Intra-patient change from baseline to days 28 and 56 in - Orthostatic/autonomic dysfunction measured by the PST - Physical exercise capacity, measured as the change in number of repetitions during the 1-min sit-to-stand test (1MSTST)
Compare the impact of PCS treatment with IMU-838 to control on autonomic function and physical activity parameters	Intra-patient change from baseline to days 28 and 56 in - Physical activity parameters: global activity (steps/24 h), wear time (min), time spent in physical activity (min), resting (bpm) and activity heart rate (bpm) - Autonomic function parameters: heart rate turbulence, nocturnal heart rate, nocturnal respiratory rate, expiration-triggered sinus arrhythmia, baroreflex sensitivity, frequency of spontaneous ectopic beats measured by use of mhealth devices (c-med alpha, CE medical device, Cosinuss GmbH, Munich, Germany, and SaniQ, CE medical device, Qurasoft GmbH, Koblenz, Germany)
Exploratory	
Identification of biomarkers to identify and pre-select patients who could benefit from the intervention	Blood, fecal biomarkers, and Imaging biomarkers
Safety	
To evaluate safety and tolerability of IMU-838	Type, frequency and severity of AEs and SAEs from day 1 to day 84

*1MSTST* 1-min sit-to-stand test, *cMRI* cerebral magnetic resonance imaging, *FSS* Fatigue Severity Scale, *GAD-7* Generalized Anxiety Disorder Scale, *mMRC* modified Medical Research Council Dyspnea Scale, *MoCA* Montreal-Cognitive-Assessment, *PEM* post exertional malaise, *PHQ* patient health questionnaire, *PROM* patient-reported outcome measures, *PST* passive standing test, *(S)AE* (serious) adverse event, *SF-36-PF* Short Form-36 Physical Function, *IMU-838* vidofludimus calcium

### Participant timeline {13}

A detailed flowchart for RAPID_REVIVE is provided in Table 3. The schedule of assessment lists all of the assessments and indicates with an “X” when they have to be performed. Screening evaluations have to be performed within 7 days prior to randomization. During the course of the trial, visits and test procedures should occur on schedule whenever possible; after day 1, visits that occur ± 3 days from the scheduled date will not constitute any protocol deviation. Two visits, days 14 and 84, are held as Videoconference visits using a digital health application (SaniQ) or phone, alternatively. After being informed about the trial and after giving their written informed consent, patients have to undergo the examinations listed in Table 3 prior to randomization. All women of childbearing potential must undergo a pregnancy test. In addition, the following parameters are assessed at screening: differential blood count, serum creatinine, blood urea nitrogen, blood uric acid, GOT, GPT, AP, gGT, total bilirubin, indirect bilirubin, amylase, lipase, glucose, triglycerides, tuberculosis interferon gamma release assay (TBC IGRA). Results of examinations routinely performed due to PCS are accepted, if they were done within 2 weeks prior to randomization. Patients must meet all inclusion criteria and none of the exclusion criteria to be considered eligible. The randomization and subsequent allocation of participants to treatments will take place directly after eligibility screening. Randomization is performed in the secuTrial® database in a 1:1 randomization ratio during an initialization phase thereafter using a response-adaptive randomization procedure. Following inclusion in the trial (day − 7), the patients should visit the trial site on days 1, 28, and 56. On days 14 and 84, the patients will be contacted via a video-conference or phone call. On day 1, patients come to the trial site, in order to receive and initiate the IMP. Patients are treated for 56 days. A cerebral magnetic resonance imaging (cMRI) is performed in a subset of patients at baseline.

**Table 3:**
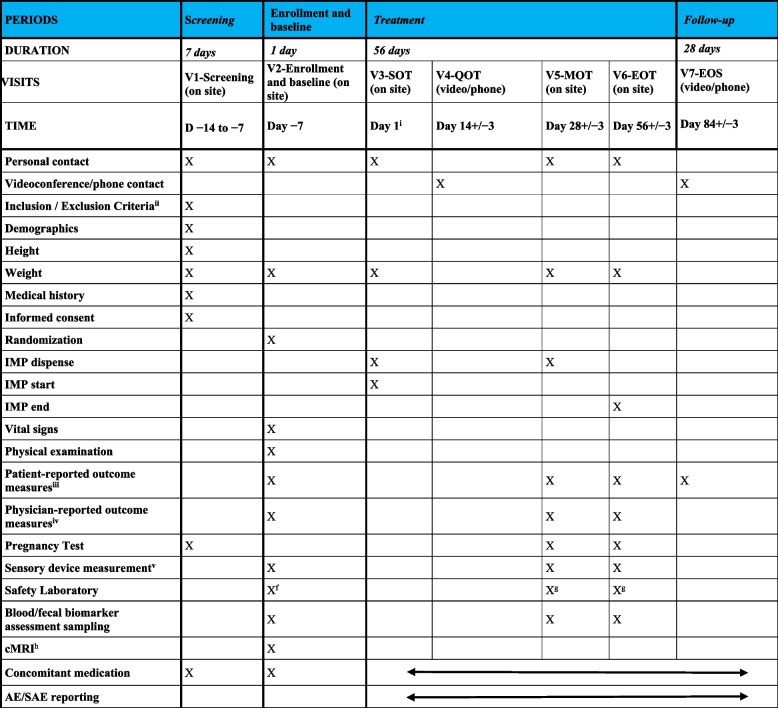
Visit schedule and assessments RAPID_REVIVE

^i^Initiation of treatment with the IMP is defined as day 1 (V3-SOT) after completion of sensory device measurements. V3-SOT may be delayed for max. 7 days after completion of sensory device measurements ^ii^The following parameters will be assessed at screening: differential blood count, serum creatinine, blood urea nitrogen, blood uric acid, GOT, GPT, AP, gGT, total bilirubin, indirect bilirubin, amylase, lipase, glucose, triglycerides, tuberculosis interferon gamma release assay (TBC IGRA). In addition, the following assessments will be performed to assess inclusion criteria: FSS, mMRC, PST, and MoCa ^iii^ Includes SF-36, FSS, GAD-7, mMRC, PEM Score, PHQ-9, PHQ-15, PHQ-stress (FSS and mMRC are not required at V2-Enrollment and Baseline) ^iv^ Includes 1MSTS, PST, MoCA (MoCA and PST are not required at V2-Enrollment and Baseline) ^v^ Measurement using the Garmin Vivosmart 5HEALTH (Qurasoft GmbH) with the SaniQ Integration software and the c-med alpha (ear sensor) and the cosinuss Health Platform (Cosinuss GmbH) over a duration of 7 days each. After enrollment at the study site, patients will be instructed on how to use the sensory devices and the first 7 days of measurement will be initiated. Measurements need to be completed before SOT Visit C2 on day 1. The second and third period of sensory device measurements will start between day 19 and day 21 to be completed before Visit C3 at day 28 assessment and between day 47 and day 49 to be completed before Visit C4 at day 56 EOT assessment ^f^ Only necessary if Screening and Enrollment/Baseline visit are more than 48 h apart. Differential blood count only ^g^ Differential blood count, serum creatinine, blood urea nitrogen, blood uric acid, GOT, GPT, AP, gGT, total bilirubin, indirect bilirubin, amylase, lipase, glucose, triglycerides ^h^ cMRI will be performed at the Enrollment Visit for a subgroup of 100 patients. For organizational reasons, regular Enrollment Visit assessments and the cMRI appointment may not always be feasible to arrange on the same date. Therefore, the cMRI may be performed until day 7. If the sensory devices have already been placed on the patient, they may have to be removed for the duration of the cMRI, but should be placed back on afterward

### Sample size {14}

In the setting of adaptive platform trials, sample size planning is done by extensive simulations to ensure the robustness of the trials under various deviations from analysis assumptions.

At this preliminary stage and given the novelty of PCS as a disease entity, only core characteristics of a generic sample size plan are given. As the precise effect size of IMU-838 in patients with PCS is unknown, the calculation for the starting sample size of this APT is based on the assumption that an improvement in terms of a standardized mean difference (Cohen’s *d*) of about 0.4 in the primary endpoint would be considered clinically meaningful. The sample size calculation is thus based on the comparison of 2 means of score outcomes of the SF-36-PF (primary endpoint), using a *t*-test (although a more complex and more powerful analysis model will be used as primary analysis). With 1:1 allocation, a sample size of *n* = 150 patients per group yields a power of 1 − *β* = 0.90 given a standardized mean difference (Cohen’s *d*) of 0.376. To adjust for a potential drop-out rate of 20% overall, another 38 patients need to be randomized per group. Thus, the total sample size across the two groups is 376 patients. If feasible, given funding period and resources, larger sample sizes are aimed for.

Calculations were performed using nQuery 9—Power and Sample Size for Group Sequential Trails (version 9.2.1.0).

### Recruitment {15}

Patient recruitment is supported by the already established, central trial infrastructures of the 3 NAPKON platforms. A recent feasibility inquiry identified more than 1000 patients with shortness of breath, more than 2000 patients with acute or persistent neurological impairment like fatigue or brain fog, and more than 2000 patients with gastrointestinal or cardiovascular symptoms or body aches in the NAPKON-SUEP and NAPKON-POP cohorts by March 2023. A minimum of 1500 patients suffered from at least 2 PCS symptoms of moderate or severe intensity. These patients had already given their consent to be re-contacted as part of the initial NAPKON informed consent. Apart from these patients, further potential trial participants may be identified through the PCS ambulatory care structures established at the different trial sites. Each site alone has seen at least 500 patients with PCS over the last 2 years. These patients may be contacted and screened through these established structures. Finally, the project partners representing patient-related concerns also intend to use their extensive networks, including access to a large community organized in social media platforms and a primary care physician network in order to identify further patients that may be suitable for participation in the trial.

## Assignment of interventions: allocation

### Sequence generation {16a}, concealment mechanism {16b}, implementation {16c}

The randomization code was generated by a statistician and provided to data management for implementation in secuTrial®. Patients are randomized using block randomization stratified by center in a 1:1 randomization ratio during an initialization phase to minimize the risk of extreme imbalance resulting in some high variable treatment effect estimates early in the trial. The initiation phase will be completed once the first 150 patients reach day 56. Following the initiation phase, patients will be allocated to the treatment arms using a response-adaptive randomization procedure to favor the more effective treatment arm, while maintaining a minimum power. The randomization ratio will be adapted at regular intervals based on the outcomes observed up to that point.

The randomization and subsequent allocation of participants to treatments takes place directly after eligibility screening. Randomization is performed in the secuTrial® database by on-site study personnel.

## Assignment of interventions: blinding

### Who will be blinded {17a}

RAPID_REVIVE is a double-blind trial, neither the patients nor the investigator knows which treatment participants are receiving. The trial statistician is blinded until the end of the recruitment. After the end of the recruitment period, he will perform a blind review of data, i.e., assessment and checking for consistency and plausibility without information on the randomized treatment for each patient. Then he will be unblinded for the final analysis.

### Procedure for unblinding if needed {17b}

As a matter of principle, unblinding in clinical trials is only performed after closure of the database for the final analysis. However, the coding system for the IMPs includes a mechanism that permits rapid identification of the product(s) in case of a medical emergency, but does not permit undetectable breaks of the blinding (ICH-GCP 5.13.4).

Any of the following can be reasons for premature unblinding:

- In emergency situations, if it is necessary for the trial patient’s safety, i.e., if the further treatment depends on the knowledge of the IMP.

To allow emergency unblinding, the investigator is supplied with a set of emergency envelopes, i.e., a sealed envelope for each treated trial patient that, if opened, reveals the treatment assigned to that patient. If emergency unblinding of a patient is necessary, the investigator will open the emergency envelope (based on the treatment/randomization/medication number) and has to enter date and time as well as their name and signature on the unblinding form contained in the envelope. The investigator also has to fax the form to the representative of the sponsor immediately.

- In the event of accidental administration of the IMP to a person who is not a trial patient.
- In the event of administration of an incorrect dose, in particular overdose of IMP which might put the patient at risk.

The decision of whether unblinding is necessary lies with the investigator. If possible, the sponsor/coordinating investigator should be consulted first.

- In the event of a SUSAR.

In case of a SUSAR, unblinding is performed by the person responsible for the pharmacovigilance/safety management in the trial. The blinding should be maintained for persons responsible for the ongoing conduct of the trial.

## Data collection and management

### Plans for assessment and collection of outcomes {18a}

RAPID_REVIVE includes a screening period of 7 days, a double-blind treatment period of 56 days and a follow-up period of 28 days. Participants are followed up for 84 days from the day of randomization. Clinic visits are scheduled at screening, day 1 (enrollment and baseline), day 28, and day 56 to assess efficacy and safety. Videoconference visits are scheduled at days 14 and 84. Once consented, the baseline data collection of the participant takes place on the trial site. The baseline visit includes the measurement of vital signs (heart rate and blood pressure), a physical examination, a safety laboratory, and the documentation of concomitant medication. In addition, all women of childbearing potential must undergo a pregnancy test. In a subset of 100 patients, cMRI is performed. Safety laboratory and the pregnancy test are also analyzed on days 28 and 56 as designated in Table 3 visit schedule and assessment. Safety laboratory includes the following parameters: blood count, serum creatinine, blood urea nitrogen, blood uric acid, GOT, GPT, AP, gGT, total bilirubin, indirect bilirubin, amylase, lipase, glucose, triglycerides, tuberculosis interferon gamma release assay (TBC IGRA).

Furthermore, the baseline patient-reported outcome measures (PROMs) are performed. Telemedical software (SaniQ Praxis; CE medical device, Qurasoft GmbH, Koblenz) is used to support acquisition of the PROMs as indicated below.

#### PROMs

##### Short Form-36 (SF-36 (RAND 1992))

The Short Form-36 (SF-36) questionnaire is an established and widely used health-related quality of life measure. The SF-36 comprises 8 domain scores including “physical functioning” (SF-36 PF). While SF-36 PF is used to assess the primary endpoint of this protocol, the complete SF-36 is needed for secondary endpoint assessment. The SF-36 PF asks respondents to report limitations on 10 mobility activities, such as walking specified distances, carrying groceries, and bathing or dressing and has been shown to be a useful tool to measure mobility disability. The possible scores range from 0 (worst, greatest limitation) to 100 (best). The SF-36 is reported via SaniQ.

##### Fatigue SeverityScale (FSS [31])

The FSS questionnaire contains nine statements that rate the severity of fatigue symptoms from 1 to 7 for each question. A low value (e.g., 1) indicates strong disagreement with the statement, whereas a high value (e.g., 7) indicates strong agreement. A higher score (36 or above) may indicate the patient is suffering from fatigue. 

##### GeneralizedAnxiety Disorder Scale-7 (GAD-7 [32])

The GAD-7 is a brief instrument designed to assess generalized anxiety disorder. The 7 items of the GAD-7 test the most important diagnostic criteria of Generalized Anxiety Disorder according to the DSM-IV and ICD-10 criteria. All items are answered for the period of the last 2 weeks and rated on a 4-point response scale with scores running from 0 (not at all) to 3 (nearly every day) for each question and the total score ranges from 0 to 21. A total score of 0 to 4 represents minimal anxiety; 5 to 9, mild anxiety, 10 to 14, moderate anxiety; and 15 to 21, severe anxiety.

##### Patient HealthQuestionnaire (PHQ)—modules

The PHQ is an internationally available, valid, and easy to use self-report instrument for assessing common psychiatric disorders. Severity of mental disorder symptoms such as depression, anxiety, somatization, and distress are measured with the following PHQ-modules (all are reported via SaniQ).

##### -Patient Health Questionnaire-9 [33]

The PHQ-9 is a 9-question instrument given to patients to screen for the presence and severity of depression. Patients are asked the number of days in the past 2 weeks they had experienced depressive symptoms with scores running from 0 (not at all) to 3 (nearly every day) for each question and a total score from 0 to 27.

##### - PHQ-15 [34]

The PHQ-15 covers 15 of the most prevalent DSM-IV symptoms of somatization disorder for a period of 4 weeks, each symptom score ranging from 0 (best option) to 2 (worst option). The total PHQ 15 score ranges from 0 to 30 and scores of 5, 10, and 15, represent cutoff points for low, medium, and high somatic symptom severity, respectively.

##### -PHQ-stress [35]

The PHQ-stress measures psychosocial strain during the last month by 10 items including health, work/financial, social, and traumatic stress. Ratings comprise “not at all bothered” (0), “bothered a little” (1), and “bothered a lot” (2). The total PHQ stress score ranges from 0 to 20. A total score of 0 to 4 represents minimal psychosocial strain; 5 to 9, mild psychosocial strain, 10 to 14, moderate psychosocial strain; and 15 to 21, severe psychosocial strain.

##### mMRC (Modified Medical Research Council Dyspnea Scale [36])

The mMRC is used to assess the degree of baseline functional disability due to dyspnea. Symptoms of dyspnea are rated by the patient from 0 to 4, with a higher score indicating worse breathlessness. The mMRC will be reported via SaniQ, Sect. 7.5.9.

##### Post-ExertionalMalaise (PEM) questionnaire [37]

The patient indicates the severity and frequency of 5 statements (from the DePaul Symptom Questionnaire) about post-exertional malaise over the last 6 months on a scale from 0 to 4 (a higher score indicating worse symptoms). At all visits following day 1, the period covered should not be 6 months, but the time since the last visit at which the PEM questionnaire was completed. The PEM is reported via SaniQ.

PROMs are performed furthermore at days 28, 56, and 84 (Table 3).

#### Physician-reported outcome measures

Physician-reported outcome measures are performed at baseline, day 28, and day 56, and include:

##### Montreal-CognitiveAssessment (MoCA [38])

The MoCA is a validated, internationally used rapid assessment for cognitive impairment. It is currently the best alternative to computerized tests. It assesses different cognitive domains: attention and concentration, executive functions, memory, language, visuoconstructional skills, conceptual thinking, calculations, and orientation. For the test, patients are instructed by a health professional to perform 11 different short tasks. A standardized form is used to support the assessments and document the results. The test results range between 0 and 30 points, where results of 26 and higher are considered normal. The assessment requires approximately 10 min.

##### 1-min sit-to-stand test (1MSTST)

The 1MSTST quantifies exercise capacity. It typically involves an armless chair and the performance of as many sit-to-stand actions as possible in 1 min without using the upper limbs. The number of repetitions is measured.

##### Passive standingtest (PST [39])

An automated blood pressure cuff is placed on the right upper limb, recording blood pressure and HR at 1-min intervals throughout the test. After 5 min of supine posture, the patient stands with their heels 2–6 in. away from the wall and with the upper back leaning comfortably against the wall. The patient remains in this position for a maximum of 10 min and is asked to minimize movement. At the end of the 5-min supine and each minute during the PST, the blood pressure and HR are recorded; thereafter, the patient is asked about the severity of orthostatic symptoms on a 0 to 10 scale.

#### Sensory device measurement

Intra-patient change in autonomic function and physical activity parameters are measured by the use of mhealth sensory devices (c-med alpha, CE medical device, Cosinuss GmbH, Munich, Germany, and SaniQ, CE medical device, Qurasoft GmbH, Koblenz, Germany). The following measurements are recorded by the respective devices:

- SaniQ + smartwatch vivosmart 5: global activity (steps/24 h), wear time (min), time spent in physical activity (min), resting (bpm), and activity heart rate (bpm),
- Cosinuss: heart rate deceleration, heart rate turbulence, nocturnal heart rate, nocturnal respiratory rate, expiration-triggered sinus arrhythmia, baroreflex sensitivity, frequency of spontaneous ectopic beats.

Measurements take place at three different times during the study, with each measurement period lasting 7 consecutive days (see Table 3):

- Measurement Period 1 at baseline: After consent, measurement needs to be initiated between day − 10 and − 7 and completed until day 1.
- Measurement Period 2 at MOT: Measurement starts between day 19 and 21 and lasts for 7 days.
- Measurement Period 3 at EOT: Measurement starts between day 47 and 49 and lasts for 7 days.

Primary analysis of the data acquired by the two digital sensors and their connected software is performed by the MRI (Cosinuss ear sensor data) and UKGM team (smartwatch data), respectively. This includes the transformation of timeline-data to numerical data of the autonomic function and physical activity parameters (parameters described in Outcomes, Key secondary endpoint 4.) The processed data are then exported into the central trial statistics (secuTrial®) from the UKGM team and the MRI team respectively.

#### Biomarker assessments

The NUM Biosample Core Unit (NUM-BCU) stores the samples for biomarker analyses, which are collected at certain intervals from the respective trial sites, centrally in Hanover or Bielefeld. Initially, samples are collected, processed, and stored locally at the trial site following the (Standard Operating Procedures) SOPs of the NUM-BCU. These processes are documented in the central biosample system (NUM-LIMS).

NUM-BCU will ship probes to the central analysis laboratories.

An in-depth biomarker assessment will be performed for all baseline samples. Samples from further visits (day 28 and day 56, see Table 3) will be stored for potential future analysis.

Two central laboratories (Goethe University Frankfurt, Charité – Universitätsmedizin Berlin) will perform biomarker assessments. Analysis results will be documented in the central biosample system (NUM-LIMS).

At the laboratory in Frankfurt, the following parameters will be assessed:

- SARS-CoV-2 neutralizing antibodies (serum)
- EBV and CMV serology (serum)
- Detection of EBV by PCR (DNA from blood)
- Detection of SARS-CoV-2 by RT-PCR (DNA from feces)

At the laboratory in Berlin, the following parameters will be assessed:

- Cytokines and chemokines (e.g., CRP, type I IFN, IL-4, IL-6, IL-1b, IL-8, IL-10, TNF, IP-10, MCP-1, MIP-3b; serum, heparin plasma, EDTA blood)
- Antibodies and autoantibodies (e.g., anti-type I IFN, anti-GPCRs G-protein-coupled receptors; serum and heparin plasma)
- Storage for later analysis: serum for proteomics
- Cytometry (CyTOF; heparin blood)
- Functional analyses of immune cell subsets (PBMCs from heparin plasma)
- Storage for later analysis: scRNAseq (PBMCs from heparin plasma)
- Complement cleavage products (EDTA plasma)
- Storage for later analysis: plasma for proteomics (EDTA plasma)

### Plans to promote participant retention and complete follow-up {18b}

Measures to increase any patient’s inclination to complete the study always address the patient directly. Patients suffering from PCS frequently report a perceived lack of recognition by the public and health care and scarce acknowledgement of the setback they experience from their health condition. Therefore, it is necessary to take the patients’ concerns seriously and particularly important to guide and give participation by the trial staff. In case of no perceived amelioration, the patient needs to be encouraged to complete the trial anyhow. Also, the telemetric and biometric devices should function smoothly and pose no annoyance for the user, and if, help should be offered from the trial site. The recruitment will take into consideration that during the roughly 3 months on the trial, visits might interfere with holidays during the festive season.

In the case trial treatment of a patient has been stopped prematurely, further follow-up visits (EOS) and the assessment of the trial endpoints are essential to enable an analysis of the full analysis set according to the intention-to-treat principle. Further visits, follow-up, and documentation should always be striven for/ensured in this case. This includes the follow-up of AEs, the time of termination, the results available at that time and, if known, the documentation of the termination of treatment in the eCRF and in the medical record, giving reasons, a final examination, and documentation according to the protocol (if possible).

### Data management {19}

The investigator records the participation in the trial, the frequency of the trial visits, the relevant medical data, the concomitant treatment, and the occurrence of adverse events in the medical record of each trial patient. An electronic data capture (EDC, secuTrial®) system is used in this trial (called eCRF). All data collected during the trial are entered in the trial-specific e-forms by the responsible investigator, or designated person, as timely as possible. Data entry and data corrections on e-forms are automatically tracked in the audit trail created by the EDC system. Data corrections in the eCRF due to queries are performed by the responsible investigator, or designated person, as timely as possible.

Data management is performed with secuTrial® which is developed, validated, and maintained by interactive Systems GmbH. The database is installed on servers of the University Medical Center Göttingen (UMG) and backed up daily. Daily backups are saved for 14 days. Details on data management (procedures, responsibilities, data corrections, if any, which may be made by data management staff themselves, etc.) are described in a data management plan prior to the trial. Before any data entry is performed, the trial database will be validated and undergo a user-test. An audit trail is created to provide an electronic record of which data were entered or subsequently changed, by whom and when. R- or SAS software is used to review the data for completeness, consistency, and plausibility in cases a direct implementation is not possible. Upon discovering inconsistencies or implausible data, queries are sent to the investigator for review. After database deactivation a copy of the database will be password encrypted and archived for 25 years within the UMG. If consent is given, long-term storage of data for secondary use is provided in a separate research database.

### Confidentiality {27}

The investigator must ensure anonymity of the patients; patients must not be identified by names in any documents submitted to sponsor. Signed informed consent forms (ICF) and patient enrollment log must be kept strictly confidential to enable patient identification at the site.

All trial-related information is stored securely at the trial site. All participant information are stored in locked file cabinets in areas with limited access. All laboratory specimens, reports, data collection, process, and administrative forms are identified by a coded identification number only, to maintain participant confidentiality.

Identity management, pseudonymization of data, and informed consent management are handled by the independent Trusted Third Party (TTP). Imaging data are handled through a centralized Digital Imaging and Communications in Medicine (DICOM) administrative system (DIMA). The collection of bio samples are performed in accordance with SOPs generated by the NUKLEUS Biobanking Core Unit (BCU).

### Plans for collection, laboratory evaluation and storage of biological specimens for genetic or molecular analysis in this trial/future use {33}

Samples obtained for biomarker assessments that are initially stored at the trial site are shipped to central laboratories in the course of the study. Remaining material will be stored for the biobank for secondary use. Secondary use refers to medical research aimed at improving the prevention, detection, and treatment of diseases. As part of secondary use, further molecular and genetic analysis can be carried out on the samples. Patients are informed about this in the consent for secondary use. See also section “Additional consent provisions for collection and use of participant data and biological specimens {26b}”.

## Statistical methods

### Statistical methods for primary and secondary outcomes {20a}

Before the start of the final analysis of the first intervention (RAPID_REVIVE) included in the platform trial, a statistical analysis plan (SAP) that encompasses the entire platform study will be prepared for the master protocol. The SAP will be completed during the “blind review” of the data, at the latest. This blind review, i.e., assessment and checking for consistency and plausibility of data, will be performed after the end of the recruitment period without information on the randomized treatment for each patient. The analysis of the primary endpoint is generic to all interventions of the platform trial including RAPID_REVIVE and is also described in the master protocol. The analysis of secondary endpoints is specific for the ISAs and clarified here for RAPID_REVIVE.

### Statistical methods for primary outcome

The primary endpoint of the RAPID platform trial is physical function measured by SF-36-PF at day 56, a continuous variable ranging from 0 to 100 with smaller values indicating greater limitations. The primary outcomes in the strata will be analyzed using mixed models for repeated measures (MMRM) approaches, i.e., Gaussian linear models for repeated measures with treatment, center, time, center, and treatment-by-time interactions as factors, and baseline measurements as covariate. The error terms are assumed to follow a multivariate normal distribution with unstructured covariance. Least squares mean changes from baseline to day 56 will be reported for both groups with 95% confidence interval (CI) as well as the difference between the least squares treatment group means with 95% CI and *p*-value testing the null hypothesis of no treatment effect. Although the models described are robust to a certain extent to missing data, sensitivity analyses will be performed as supporting analyses including reference-based multiple imputation and shared random effects models. Although the response adaptive randomization procedure maintains consistency and asymptotic normality of estimators under simple conditions [40], we will perform a re-randomization test as sensitivity analysis.

### Statistical methods for secondary outcomes

Secondary endpoints SF-36, FSS, PHQ-9, GAD-7, PHQ-15, PHQ-stress, MoCA, mMRC, PEM, PST, 1MSTST, and continuous health activity parameters of RAPID_REVIVE are analyzed analogously to the primary endpoint described above with additional least square mean comparisons between at days 28 and 84. Likewise, changes in biomarkers from baseline to day 56, such as cytokines and chemokines will also be evaluated analogously to the primary endpoint.

For secondary endpoints only involving changes from baseline to day 28, a linear regression model, with treatment and center as factors, and the baseline measurement as covariates will be employed. Treatment effects will be reported as the difference between least squares means for treatment groups, accompanied by a 95% CI and a *p*-value for testing the null hypothesis of no treatment effect. All hypothesis tests are performed two-sided at 5% significance level, unless stated otherwise.

### Interim analyses {21b}

Multiple interim analyses are planned be conducted by the Data Safety Monitoring Board (DSMB) to monitor efficacy and safety of the study interventions in the master protocol RAPID. The adaptive platform trial infrastructure facilitates the ongoing evaluation of emerging therapies in terms of efficacy and safety in stratified populations. Over time the therapies included in the platform as well as the strata can change as new evidence regarding the efficacy and safety of treatments as well as the population stratification emerges from within the platform trial or outside the trial (e.g., as part of systematic reviews [41]). These adaptations of the platform will be facilitated through the regular interim analyses by the DSMB and the trial statistician [42]. The function of the DSMB is to monitor the course of the trial and if necessary to give a recommendation to the sponsor/coordinating investigator/SC for discontinuation, modification, or continuation of the trial.

Interim analysis for, e.g., futility or efficacy stopping, adaptive enrichment, or re-stratification are intervention specific and not described in the master protocol.

For RAPID_REVIVE no interim analyses for futility or efficacy stopping are planned. At regular intervals, interim data will be provided to a statistician to generate the randomization code using a response adaptive randomization procedure. The first interim look for the response-adaptive randomization procedure in RAPID_REVIVE is planned after 150 patients reach day 56.

### Methods for additional analyses (e.g., subgroup analyses) {20b}

An exploratory objective of the RAPID_REVIVE ISA is to identify blood and imaging biomarkers, as well as clinical characteristics that allow the pre-selection of patients with a high response to the interventional treatment.

A subgroup of 100 patients receive a cMRI to allow for adaptation of inclusion criteria over the course of the trial.

The cMRI parameters are

- Limbic system microstructural integrity measured as fractional anisotropy and mean diffusivity in deep gray matter structures
- Gray matter thickness in the orbitofrontal cortex, parahippocampal gyrus, insula, and anterior cingulate
- Hippocampal volume, thalamic volume, and whole brain volume assessed in a subgroup of 100 patients

For all 376 patients, a biomarker analysis is performed at baseline.

Biomarkers measured at baseline, i.e.:

- Cytokines and chemokines (Type I IFN, IL-4, IL-6, IL-1b, IL-8, IL-10, TNF, IP-10, MCP-1, MIP-3b)
- Markers of inflammation and neuronal damage (e.g., C3a, C5a, CRP, GFAP, BDNF, NF L, S100A8/A9)
- Viral proteins and serology (SARS-CoV-2: neutralizing antibodies and DNA from feces; EBV DNA from blood)
- Autoantibodies (e.g., anti-type I IFN, anti-G-protein-coupled receptors)
- Immune cell composition and activation

For this purpose, the baseline cMRI parameters and their interaction with treatment will be included in the regression models described above for the primary and key secondary endpoints. An analogous analysis is performed for biomarkers measured at baseline.

### Methods in analysis to handle protocol non-adherence and any statistical methods to handle missing data {20c}

Efficacy analyses will be performed primarily in the full analysis set (FAS) according to the intention-to-treat (ITT) principle. This means that the patients will be analyzed in the treatment arms to which they were randomized, irrespective of whether they refused or discontinued the treatment or whether other protocol violations are revealed.

The per-protocol (PP) population is a subset of the FAS and is defined as the group of patients who had no major protocol violations, received a predefined minimum dose of the treatment and underwent the examinations required for the assessment of the endpoints at relevant, predefined times. The analysis of the PP population will be performed for the purpose of a sensitivity analysis.

Safety analyses will be performed in the safety analysis set. Patients in the safety population are analyzed as belonging to the treatment arm defined by treatment received.

### Plans to give access to the full protocol, participant-leveldata, and statistical code {31c}

This protocol is posted in the EU Clinical Trial Register (CTIS). In addition, upon trial completion, the results of this trial will be submitted for publication and/or posted in a publicly accessible database of clinical trial results irrespective of the results of the trial.

The key aspects and underlying rules and measures regarding the transfer of data and biospecimen for secondary use projects through the University Medicine Network (NUM) Use and Access Process are described in the separate ICF for secondary use (Attachment 3).

## Oversight and monitoring

### Composition of the coordinating center and trial steering committee {5d}

The sponsor of RAPID is the Goethe University Frankfurt represented by the principal investigator (PI). The coordinating study center of RAPID_REVIVE is the Infectiology focus at the University Hospital Frankfurt. The clinical trial coordination is supported by the Clinical Trials Unit (CTU), University Medical Center, Göttingen (UMG). The UMG is also responsible for Monitoring, Pharmacovigilance, Data management, Randomization, and Biostatistics of RAPID.

The governance of RAPID will be key to ensuring its smooth set-up and conduct. The governance consists of.

- The scientific steering committee (SC),
- The data safety monitoring board (DSMB), and.
- The statistical analysis center (SAC).

#### Steering commitee

A trial related steering committee (SC) has appointed by the sponsor prior to the start of the trial. The SC is responsible for the management and conduct of RAPID and is composed of the two project leaders, the trial statistician, a patient representative, a member of the NAPKON SC, a representative of the Nationale Klinische Studiengruppe (NKSG) Chronic Fatigue Syndrome/Myalgic Encephalomyelitis (ME/CFS) und Post-COVID-19-Syndrom as well as a representative of a recruiting site.

The SC was involved in the development of the protocol and ensures transparent management of the trial according to the protocol through recommending and approving modifications as circumstances require. The SC reviews protocol amendments as appropriate. Together with the clinical trial team, the SC will also develop recommendations for publications of trial results including authorship rules. SC Meetings are organized by the CTU UMG.

In particular, the SC votes on the further proceedings after the DSMB has made a recommendation on adaptation of the trial.

#### Statistical Analysis Center

The Statistical Analysis Center (SAC), situated at the University Hospital Göttingen, performs all analyses related to the trial and makes them accessible to the DSMB. The SAC includes the trial statistician.

#### Patient involvement

A patient representative from Long COVID Germany, a German self-help group, is involved in the planning and conduct of RAPID_REVIVE. She was involved in the early planning process and revised the protocol and informed consent. The patient representative is also part of the steering committee as mentioned above and thus involved in all key decisions regarding the trial. Long COVID Germany also provided advice on how to improve patient recruitment.

### Composition of the data monitoring committee, its role and reporting structure {21a}

A Data Safety Monitoring Board (DSMB), composed of one statistician and two clinicians from the NUM FOSA (German: Fach- und Organspezifische Arbeitsgruppen, English: area- and organ-specific working groups), was established. One of these was appointed as the chair of the DSMB.

The function of the DSMB is to monitor the course of the trial and if necessary to give a recommendation to the sponsor/coordinating investigator/SC for discontinuation, modification, or continuation of the trial. The underlying principles for the DSMB are ethical and safety aspects for the patients. It is the task of the DSMB to examine whether the conduct of the trial is still ethically justifiable, whether security of the patients is ensured, and whether the process of the trial is acceptable. For this the DSMB has to be informed about the adherence to the protocol, the patient recruitment, and the observed adverse events. The DSMB will receive the corresponding reports at the time of the planned interim analyses. The blinded export provided by the data management is used by the trial statistician for the creation of the script for the analysis of the unblinded export. The trial statistician, who is always blinded, gives this code to the independent statistician, who uses it to analyze the unblinded export and transmits the report to the DSMB in encrypted form.

Once the pre-specified threshold levels of probability for superiority, inferiority, or equivalence have been reached within a specific APT subprotocol, the Statistical Analyses Center will communicate this event to the DSMB. The DSMB will then review and discuss the reported results. Finally, it will give a recommendation on adaptation of the trial to the SC, which will then vote on the further proceedings. The composition and responsibilities of the DSMB, the structure and procedures of its meetings, and its relationship to other key trial team members (SC), are laid down in an DSMB charter.

### Adverse event reporting and harms {22}

#### Adverse events

An AE is any untoward medical occurrence in a patient or clinical trial subject administered a medicinal product and which does not necessarily have a causal relationship with this treatment. An AE can therefore be any unfavorable and unintended sign (including an abnormal laboratory finding), symptom, or disease temporally associated with the use of a medicinal product, whether or not considered related to the medicinal product.

Over the whole period of patient trial participation, all AEs spontaneously reported by the patient or observed by the investigator must be documented in the medical record and on the designated case report form (AE eCRF page). All AEs must be captured whether regarded as trial related or not.

AEs must be described by diagnosis or, if an underlying diagnosis is not known, by symptoms or medically significant laboratory or instrumental abnormalities. The AEs should be documented as described below.

Pre-existing conditions are not to be considered an AE, but need to be documented in the medical history section of the eCRF. Worsening of a pre-existing condition is considered an AE and needs to be documented.

All AEs, no matter how intense, are to be followed up by the investigator until resolved or judged no longer clinically relevant.

#### AEs of special interest (AESI)

The following AEs are defined as AESI if these events are different from any pre-existing conditions or a result from known conditions:

- RBC urine positive (as defined below), at least of moderate intensity
- Hematuria (as defined below)
- Retroperitoneal colicky pain in connection with suspected or confirmed nephrolithiasis

Severity is a clinical observation and describes the intensity of the event. Because of the lack of widely accepted categorization, the severity of hematuria is classified as follows:

- Mild: Asymptomatic hematuria; clinical or diagnostic observations only.
- Moderate: Symptomatic hematuria, e.g., with moderate flank pain (and including short-term (less than 24 h), standard dose therapy with oral nonsteroidal anti-inflammatory drugs, oral acetaminophen, or oral aspirin), interfering with but not limiting activities of daily living.
- Severe: Gross or macrohematuria. Any hematuria with severe flank pain limiting activities of daily living. Any hematuria requiring additional treatment (e.g., oral anti-emetics or muscle relaxants, around-the-clock narcotic analgesics, use of narcotics, or any intravenous treatment) or procedures for maintaining adequate urinary flow (e.g., urinary catheter or bladder irrigation).

The evaluation of RBC in urine will be solely based on findings from microscopic examinations of urinary sediment and not from dipstick reading only. Therefore, all conspicuous dipstick readings will be followed up by a microscopic examination of urinary sediment. All findings of RBC in urine per high-powered field will be listed as urinalysis abnormalities but not as an AE if assessed by the investigator as not clinically significant. The investigator will also assess any increased RBC in urine as not clinically significant if there are more likely alternatives to explain this finding. The following alternative explanations of RBC in urine high will be considered:

- The urine sample was not properly collected (random midstream clean-catch collection) or evidence of contamination (e.g., presence of bacteria or an unusual number of epithelial cells in urine sediment not explained by other conditions).
- Evidence of infection not considered secondary to drug-induced damage.
- Likely benign causes, such as menstruation, vigorous exercise, viral illness, trauma, and infection.

If any finding of “RBC in urine high” is assessed by the investigator as clinically significant (causing clinical action, at least retesting), this finding will be reported as the AE “RBC urine positive.”

Any occurrence of RBC urine positive will only be defined as the AE “hematuria” if at least one of the following 2 conditions is met:

- ≥ 5 RBCs per high-powered field were found in at least 2 consecutive, properly collected urinalysis specimens and/or
- The finding of RBC urine positive had diagnostic or therapeutic consequences.

AESIs will be reported as SAEs as described below.

#### Documentation of AEs

Adverse events have to be monitored and documented in the eCRF over the whole period of patient trial participation. The following data need to be documented:

- Characterization of the event (diagnosis; if not available, symptoms)
- Onset date/date of resolution
- Severity of event (“mild, moderate, severe, life-threatening, fatal”)
- Relationship to the IMP (related/not related), the expression “related” means that there is evidence or argument to suggest a reasonable causal relationship between the event and the administration of the IMP, e.g., close temporal connection, exclusion of other causes. The assessment “not related” is appropriate if the AE is clearly or most likely explained by other causes even if a potential relationship between IMP and the AE cannot be completely excluded.
- Serious/non-serious
- Action taken with regard to IMP (continued/stopped/interrupted)
- Outcome of AE

#### Pregnancies

In case of pregnancy, the patient must immediately be withdrawn from the trial treatment.

Any pregnancy (female trial participant or female partner of male trial participant) that occurs during trial participation must be reported. To ensure patient safety each pregnancy must be reported to CTU Vigilance on the pregnancy reporting form within 24 h of learning of its occurrence. The reporting follows the same instructions as described for SAEs 

The pregnancy should be followed up to determine outcome, including spontaneous or voluntary termination, details of birth, and the presence/absence of any birth defects, congenital abnormalities, or maternal and new-born complications.

The details on the implementation of this requirement is described in a trial specific SAE-manual.

#### Serious adverse events

A serious adverse event (SAE) is any AE that at any dose.

- Results in death,
- Is life-threatening,
- Requires hospitalization or prolongation of existing hospitalization (excluding those for trial therapy and elective or pre-planned treatment/surgery)
- Results in persistent or significant disability or incapacity,
- Is a congenital anomaly or birth defect,
- Other medically important event: events that may jeopardize the patient or may require an intervention to prevent one of the above characteristics/consequences. Such events should also be considered “serious” in accordance with the definition and have to be reported as SAEs.
- Cases of misuse and abuse of IMP should also be reported as SAEs.

Please note:

- The term “life-threatening” in the definition of “serious” refers to an event in which the patient was at risk of death at the time of the event; it does not refer to an event which hypothetically might have caused death if it were more severe.

#### Documentation and reporting of SAEs

All SAEs that occur starting from signature of the informed consent form until the end of patient’s follow-up period have to be entered into the eCRF *within 24 hafter investigator awareness*. The documented SAEs will be sent to the Sponsor via the reporting function of the eCRF. In case of failure of the electronic reporting function of the eCRF, the SAE paper form must be completed and sent via fax or e-mail *within 24 h* after investigator awareness to the Vigilance of the CTU UMG.

If new information including outcome becomes available or, e.g., relationship to IMP(s) is reconsidered, a SAE follow-up report must be sent within 24 h using the same procedure as for transmitting the initial SAE report. The reporting and processing of the SAEs is described in detail in trial specific SAE-manual.

#### Specific protocol exceptions to SAE reporting

Events not to be reported as SAEs are hospitalizations for the following:

- Routine treatment or monitoring of the studied indication, not associated with any deterioration in condition.
- Treatment, which was elective or pre-planned, for a pre-existing condition that is unrelated to the indication under study and did not worsen.
- Treatment on an emergency, outpatient basis for an event not fulfilling any of the definitions of SAE given above and not resulting in hospital admission.

### Frequency and plans for auditing trial conduct {23}

Monitoring and source data verification are the important parts of the auditing trial conduct independent from investigators and the sponsor.

#### Monitoring

Monitoring is performed by the Clinical Research Associates (CRAs) of UMG CTU. Risk-based monitoring will be done according to ICH-GCP E6 and SOPs to verify that patients’ rights and wellbeing are protected, reported trial data are accurate, complete, and verifiable from source documents and that the trial is conducted in compliance with the currently approved protocol or, if applicable, amendment, with ICH-GCP and with the applicable regulatory.

The investigator will accept monitoring visits before, during, and after the clinical trial. Prior to the trial, a site initiation visit at each site OR at an investigators meeting is conducted in order to train and introduce the investigators and their staff to the trial protocol, essential documents, handling of IMP and related trial-specific procedures, ICH-GCP, and national/local regulatory requirements.

During the trial, the CRA visits the site regularly OR once a year depending on the recruitment rate and quality of data. During these on-site visits, the CRA verifies that the trial is conducted according to the trial protocol, trial-specific procedures, ICH-GCP, and national/local regulatory requirements. The presence of signed informed consents, eligibility of patients, documentation of primary endpoint, handling of IMP, and documentation/reporting of safety data (e.g., AE/SAE) will be verified by the CRA.

#### Source data verification (SDV)

The CRA also performs source data verification (SDV) and drug accountability checks to ensure that the clinical trial data which are recorded in the source data and eCRFs are complete and accurate. Extent of source data verification and monitor visit frequency will be adapted for individual sites in case of lack of data quality or a high number of protocol violations. All trial-specific monitoring procedures, monitoring visit frequency, and extent of SDV will be predefined in a trial-specific monitoring manual. The investigator must maintain source documents for each patient in the trial, consisting of case and visit notes (hospital or clinic medical records) containing demographic and medical information, laboratory data, electrocardiograms (ECG), and the results of any other tests or assessments. All information recorded in eCRFs must be traceable to source documents in the patient’s file. The investigator must also keep the original signed informed consent form (a signed copy is given to the patient). The investigator must give the CRA access to all relevant source documents to confirm their consistency with the eCRF entries. Source data as defined by ICH-GCP include original documents, data, and records such as hospital records, clinical and office charts, laboratory notes, memoranda, patients’ diaries or evaluation checklists, pharmacy dispensing records, recorded data from automated instruments, copies or transcriptions certified after verification as being accurate copies, microfiches, photographic negatives, microfilm or magnetic media, X-rays, and records kept at the pharmacy, at the laboratories and at medico-technical departments involved in the clinical trial.

### Plans for communicating important protocol amendments to relevant parties (e.g., trial participants, ethical committees) {25}

Any change or addition to the protocol can only be made in a written protocol amendment that must be approved by sponsor and the Member State(s). Protocol amendments will be reviewed by the SC as appropriate.

Regardless of the need for approval of formal protocol amendments, the investigator is expected to take immediate action required for the safety of any patient included in this trial, even if this action represents a deviation from the protocol. In such cases, the sponsor has to be notified as soon as possible of this action.

Information regarding important protocol modifications will be provided in due time to further relevant parties (e.g., ethical committees, nvestigators, trial participants, trial registries, journals).

### Dissemination plans {31a}

Upon trial completion the results of this trial will be submitted for publication and/or posted in a publicly accessible database of clinical trial results irrespective of the results of the trial. Reporting guidelines will be taken into account (see www.equator-network.org), the Consolidated Standards Of Reporting Trials (CONSORT) statement will be adhered to in the preparation of papers on the results of randomized studies.

Each publication of trial results will be in mutual agreement between the coordinating investigator, the other investigators involved, and the CTU/the SC. All data collected in connection with the clinical trial will be treated in confidence by the coordinating investigator and all others involved in the trial, until publication. Interim data and final results may only be published (orally or in writing) with the agreement of the coordinating investigator and the CTU/the SC.

To achieve maximum impact of results, different channels of dissemination targeting different stakeholders and communities will be used. Since PCS is a global concern, dissemination of results will not be limited to Germany or Europe. Results of the trial will be published in a scientific peer-reviewed journal under open access conditions. In addition, information on the trial will be disseminated through social media channels and through presentation at national and international conferences of the relevant academic disciplines.

All research data collection and generation associated with the trial will follow the guiding principles of findability (F), accessibility (A), interoperability (I), and reusability (R, together: FAIR). To achieve optimum findability, studies will be registered with WHO approved trial registries (F). Data items and forms will be published on the Medical Data Models Portal (MDM Portal, https://medical-data-models.org) (F, A). Where feasible, integration of data and samples into major infrastructures, e.g., the German Biobank Node, the Zentrale Antrags- und Registerstelle (ZARS) and applicable infrastructures of the German Centers for Health Research (Deutsche Zentren der Gesundheitsforschung, DZG) is intended (F, A). Data will be stored in common database and file formats, with comprehensive codebooks explaining file and data format and rules for collection (A, I). Data shall be published under consideration of ethical principles and GDPR regulation and made available with an adequate open license to a broader scientific community (R).

## Discussion

Medical recommendations are based on high-quality evidence, ideally generated by randomized controlled trials (RCTs). Accordingly, to ensure that sufficient data is available to assess a medical situation, a number of RCTs must be conducted in which only a limited number of interventions in a single indication are evaluated. Although this is still the gold standard for gaining clinical-scientific knowledge, it is characterized by high costs, long implementation times and a lack of flexibility with regard to the integration of new findings that are obtained within and outside the respective study. Using the example of acute coronavirus disease 2019 (COVID-19), it became clear that so-called adaptive platform studies offer practicable solutions to the abovementioned problems and are indispensable in a situation in which the evidence base changes continuously and rapidly and study components have to be adapted accordingly. In Germany, the necessary overarching platform is currently being realized by the already BMBF funded NAPKON-TIP project (National Pandemic Cohort Network—Therapeutic Intervention Platform) in close cooperation with the existing structures of NAPKON and NUM (Network of University Medicine).

RAPID is the first use case to be assessed using the NAPKON-TIP platform. Despite a large number of considerations regarding treatment options for PCS and a considerable number of studies that have been initiated, very few results from randomized controlled trials are currently available. At the same time, numerous hypotheses concerning potentially effective treatment strategies are slowly emerging from ongoing research [14]. The successful completion of this platform study will add to the very limited evidence base for the treatment of PCS and thus contribute to the health of a large patient population worldwide. In addition to the intervention “antiviral treatment” offered as part of the first ISA RAPID_REVIVE and which is presented in this manuscript in detail, further interventions can be included at a later stage, providing the unique opportunity to compare different interventions in a single trial by responding very quickly to the rapidly changing evidence base for PCS in particular. As part of RAPID_REVIVE patients will also undergo cerebral imaging as well as immunological and virological biomarker assessments. This data will be used to enable stratification of patients with regard to response prediction over the course of the adaptive platform study. If such stratification is feasible, the inclusion and/or exclusion criteria will be adjusted during the study.

## Trial status

Protocol version number: V2.0, date: 13.05.2024; date of recruitment begin: 27.08.2024; the approximate date when recruitment will be completed 30.06.2025.

## Supplementary Information

Additional file 1.

Additional file 2.

Additional file 3.

## Data Availability

Data from the clinical trial will be made available to third parties via the NUM use and access rules. RAPID_REVIVE will obey Annex IV as well as Annex V of the CTR (EU) 536/2014.

## Abbreviations

1MSTST: 1-Minute sit-to-stand test
ACE2: Angiotensin converting enzyme 2 receptor
AE: Adverse event
APT: Adaptive Platform Trial
BCU: Biobanking Core Unit
BCRP: Breast Cancer Resistance Protein
BMBF: German Federal Ministry of Education and Research
CI: Confidence interval
CYP: Cytochrome
cMRI: Cerebral magnetic resonance imaging
CMV: Cytomegalovirus
CONSORT: Consolidated Standards Of Reporting Trials
COVID-19: Coronavirus Disease 2019
CRA: Clinical Research Associate (on-site monitor)
CTIS: Clinical Trials Information System
CTU: Clinical trials unit
DHODH: Dihydroorotate dehydrogenase
DICOM: Digital Imaging and Communications in Medicine
DIMA: DICOM Management System
DMARDs: Disease-modifying anti-rheumatic drugs
EBV: Epstein-Barr Virus
ECG: Electrocardiogram
eCRF: Electronic Case Report Form
EDC: Electronic Data Capture
EOS: End of study
EOT: End of treatment
FAIR: Findability, Accessibility, Interoperability and Reusability
FAS: Full Analysis Set
FSS: Fatigue Severity Scale
GAD-7: Generalized Anxiety Disorder Scale
HIV: Human immunodeficiency virus
HMA: Heads of Medicines Agencies
IB: Investigator’s Brochure
ICF: Informed consent form
ICH-GCP: ICH Topic E6: Guideline for Good Clinical Practice (GCP)
IMP: Investigational Medicinal Product
IMU-838: Vidofludimus calcium
ISA: Intervention-Specific Appendix
ITT: Intention to treat
ME/CFS: Chronic fatigue syndrome/myalgic encephalomyelitis
mMRC: Modified Medical Research Council Dyspnea Scale
MMRM: Mixed models for repeated measures
MoCA: Montreal Cognitive Assessment
MOT: Middle of treatment
NAPKON: Nationales Pandemie Kohorten Netz
NUM: Network of University Medicine
NYHA: New York Heart Association
PCS: Post COVID syndrome
PEM: Post exertional malaise
PHQ: Patient Health Questionnaire
PI: Principal Investigator
PP: Per-protocol
PROM: Patient-reported outcome measures
PST: Passive standing test
RBC: Red blood cell count
S1: Spike 1
SAE: Serious adverse event
SAP: Statistical Analysis Plan
SARS-CoV-2: Severe acute respiratory syndrome coronavirus type 2
SAC: Statistical Analysis Center
SC: Steering Committee
SDV: Source Data Verification
SF-36-PF: Short Form-36 Physical Function
SOP: Standard Operating Procedure
SUSAR: Suspected unexpected serious adverse reaction
UGT1A1: Uridine-diphosphate glucuronosyltransferase 1A1
TTP: Trusted Third Party
ULN: Upper Limit of Normal
UMG: University Medical Center Göttingen
UMG CTU: Clinical Trial Unit of the University Medical Center Göttingen
WHO: World Health Organization
