# Supplementary material for: Randomized adaptive assessment of post COVID syndrome treatments (RAPID): a study protocol for a multicenter, randomized, controlled adaptive platform trial of treatment options for Post Covid Syndrome (PCS) on patients physical function including the first intervention specific appendix RAPID_REVIVE (reducing inflammatory activity in patients with PCS)
Source: Trials. 2025 Aug 19;26:297. doi: 10.1186/s13063-025-09008-0 (PMC12366011; doi:10.1186/s13063-025-09008-0)
Supplement: Supplementary file 1 — Additional file 1. [file 13063_2025_9008_MOESM1_ESM.pdf]

Patienteninformation und Einwilligungserklärung zu einer klinischen Prüfung (Studie) mit optionalem MRT

Prüfzentrum:

xy

Hauptprüfer oder einziger Prüfer: XXX

EU trial number: 2024-511628-16-00

Name und Anschrift des Sponsors: Goethe-Universität Frankfurt, vertreten durch den Präsidenten, dieser vertreten durch [redacted]

Universitätsklinikum Frankfurt
Theodor-Stern-Kai 7
60590 Frankfurt
Tel.: [redacted]
E-Mail: [redacted]

Randomisierte Studie zur Untersuchung von Behandlungsoptionen des Post-COVID Syndroms

Randomized adaptive assessment of post COVID syndrome treatments_Reducing Inflammatory Activity in Patients with post COVID Syndrome

Prüfplancode: RAPID_REVIVE

Sehr geehrte Patientin, sehr geehrter Patient,

wir möchten Sie fragen, ob Sie bereit sind, an der nachfolgend beschriebenen klinischen Prüfung (Studie) teilzunehmen.

Klinische Prüfungen sind notwendig, um Erkenntnisse über die Wirksamkeit und Verträglichkeit von Arzneimitteln zu gewinnen oder zu erweitern. Deshalb schreibt der Gesetzgeber im Arzneimittelgesetz vor, dass neue Arzneimittel klinisch geprüft werden müssen. Die klinische Prüfung, die wir Ihnen hier vorstellen, wurde – wie es das Gesetz verlangt – von der zuständigen nationalen Behörde unter Einbeziehung der national zuständigen Ethikkommission genehmigt.

Diese klinische Prüfung wird an mehreren Orten durchgeführt; es sollen insgesamt 376 Personen daran teilnehmen. An unserem Zentrum sollen ungefähr 40 Personen an der

klinischen Prüfung teilnehmen. Die Studie wird durch den oben genannten Sponsor veranlasst und finanziert.

Ihre Teilnahme an dieser klinischen Prüfung ist freiwillig. Sie werden in diese Prüfung also nur dann einbezogen, wenn Sie dazu schriftlich Ihre Einwilligung erklären. Sofern Sie nicht an der klinischen Prüfung teilnehmen oder später aus ihr ausscheiden möchten, erwachsen Ihnen daraus keine Nachteile.

Der nachfolgende Text soll Ihnen die Ziele und den Ablauf erläutern. Der Text ist in drei Teile gegliedert:

- Kurzdarstellung der Studie.
- Teil I: Informationen zum Ablauf der klinischen Prüfung einschließlich der damit zusammenhängenden gesundheitlichen Gesichtspunkte.
- Teil II: spezifische Informationen zum Datenschutz und zu den Bioproben (Blut, Stuhl etc.).

Auf jeden Fall wird eine Prüfärztin oder ein Prüfarzt ein Aufklärungsgespräch mit Ihnen führen. Bitte zögern Sie nicht, alle Punkte anzusprechen, die Ihnen unklar sind. Sie können gerne Sätze und Abschnitte markieren, die Sie nicht verstanden haben, um sie mit dem aufklärenden Arzt zu besprechen. Sie werden danach ausreichend Bedenkzeit erhalten, um über die Teilnahme zu entscheiden.

Kurzdarstellung der Studie

Grund für die Studie: Menschen mit einem Post COVID Syndrom haben über Monate hinweg Beschwerden. Diese Beschwerden können sehr unterschiedlich sein, und die Ursachen sind noch nicht vollständig verstanden.
In dieser Studie wollen wir ein neues Medikament, IMU-838, mit dem Wirkstoff Vidofludimus Calcium (VidoCa) untersuchen.

Das getestete Prüfpräparat: Das Prüfpräparat IMU-838 wurde bisher nicht zugelassen und ist noch in der klinischen Prüfung.
Es wird seit einigen Jahren für die Behandlung von Multipler Sklerose, einer entzündlichen Hirnerkrankung, in klinischen Studien geprüft. Neuere Studienergebnisse haben gezeigt, dass IMU-838 auch gegen Viren wirken kann.
Wir möchten prüfen, ob IMU-838 in der Lage ist, den Krankheitsverlauf des Post COVID Syndroms zu verkürzen.

Studienablauf: Die Studienteilnahme für Sie dauert insgesamt knapp drei Monate.
Wenn Sie an der Studie teilnehmen, werden Sie zunächst untersucht.
Es gibt dann zwei Möglichkeiten, wie Sie behandelt werden:
1) Sie bekommen entweder über 56 Tage IMU-838 als Tablette oder
2) Sie bekommen 56 Tage ein Placebo als Tablette.
Ein Placebo sieht aus wie IMU-838, enthält aber keinen Wirkstoff. Die Entscheidung, zu welcher Gruppe Sie eingeteilt werden, erfolgt per Zufall (Randomisierung), Ihre Ärztinnen und Ärzte haben hierauf keinen Einfluss und wissen nicht, zu welcher Gruppe Sie zugeordnet werden.
Wir beobachten bis zum 84. Tag Ihren Gesundheitszustand. Sie werden dazu mehrere Fragebögen ausfüllen, Blut- und Stuhlproben werden gewonnen. Sie tragen einige Tage einen Ohrsensor und eine Armbanduhr, die Ihre Herzfrequenz, Atemfrequenz, Sauerstoffsättigung, Temperatur und Bewegung messen. Wenn Sie dazu einwilligen, wird zusätzlich ein MRT (Magnetresonanztomographie) vom Kopf gemacht.

Möglicher Nutzen: Aufgrund der bisherigen Forschungsergebnisse erhoffen sich Sponsor und Prüfarzt, dass die Behandlung mit IMU-838 Ihren Krankheitsverlauf verkürzt.

Das ist bisher jedoch nicht nachgewiesen. Es kann deshalb sein, dass diese Behandlung für Sie keinen Nutzen haben wird. Dies ist auch der Fall, wenn Sie in die Placebo-Gruppe eingeteilt werden.

Risiken und Belastungen: Die Teilnahme an einer Studie ist mit Risiken und Belastungen verbunden.

• IMU-838 kann Nebenwirkungen haben, die über die Risiken Ihrer Standardtherapie hinausgehen. Insgesamt wurden bisher jedoch keine relevanten Nebenwirkungen festgestellt. Nur bei der Einnahme von hohen Dosen von IMU-838 kam es vereinzelt zu Blut im Urin. Daneben sind leichtere Nebenwirkungen zu erwarten. In seltenen Fällen können aber auch bislang unbekannte andere schwere Nebenwirkungen auftreten.

• Die studienbedingten Untersuchungen sind regelmäßig mit Belastungen und ggf. Beeinträchtigungen verbunden. Darüber hinaus haben sie vor allem folgende Risiken: Im Verlauf der klinischen Prüfung wird Ihnen dreimal Blut abgenommen. Dies kann Schmerzen und sehr selten bleibende Schäden verursachen. Das MRT (Kernspin) des Kopfes kann als unangenehm empfunden werden, da Sie in einer geschlossenen Röhre liegen und die Untersuchung relativ laut ist. Der Ohrsensor oder die Armbanduhr können als störend empfunden werden.

Eine ausführlichere Angabe der Risiken und Belastungen finden Sie im Teil I unter Punkt 5. Der Prüfarzt wird mit Ihnen darüber ausführlich sprechen.

Freiwilligkeit: Es ist Ihre freie Entscheidung, ob Sie an dieser Studie teilnehmen möchten oder nicht. Sie können jederzeit NEIN sagen, sofort oder auch später. Sie brauchen dafür keine Gründe anzugeben und haben keine Nachteile in Ihrer weiteren medizinischen Versorgung.

Zusätzlich zur schriftlichen Information werden Sie mündlich aufgeklärt. Fragen Sie den Prüfarzt, wenn Sie etwas nicht verstehen. Sie haben anschließend ausreichend Bedenkzeit, um sich für oder gegen eine Teilnahme zu entscheiden. Wenn Sie sich für die Teilnahme entscheiden, unterschreiben Sie bitte die Einwilligungserklärung.

Inhaltsverzeichnis

Teil I: Informationen zum Ablauf der klinischen Prüfung und zu gesundheitlichen Aspekten 6
- I. 1. Warum und mit welchem Prüfpräparat wird diese Prüfung durchgeführt? 6
- I. 2. Erhalte ich das Prüfpräparat auf jeden Fall? 6
- I. 3. Wie ist der Ablauf der Studie und was ist bei der Teilnahme zu beachten? 7
- I. 4. Welchen persönlichen Nutzen habe ich von der Teilnahme an der Studie? 9
- I. 5. Welche gesundheitlichen Risiken und Belastungen sind mit der Teilnahme an der Studie verbunden? 10
  - a) Risiken durch das Prüfmedikament IMU-838 10
  - b) Risiken durch studienbedingte Maßnahmen 11
- I. 6. Welche anderen Behandlungsmöglichkeiten gibt es außerhalb der Studie? 12
- I. 7. Wer darf an dieser klinischen Prüfung nicht teilnehmen? 12
- I. 8. Entstehen für mich Kosten durch die Teilnahme an der klinischen Prüfung? Erhalte ich eine Aufwandsentschädigung? 13
- I. 9. Bin ich während der klinischen Prüfung versichert? 13
- I. 10. Werden mir neue Erkenntnisse zu der klinischen Prüfung mitgeteilt? 15
- I. 11. Wer entscheidet, ob ich aus der klinischen Prüfung ausscheide? 15
- I. 12. An wen wende ich mich bei weiteren Fragen? 15

Teil II: Informationen zum Datenschutz und zu den Bioproben 17
- II. 1. Was geschieht mit den über mich erhobenen Daten? 17
  - a) Allgemeine Informationen 17
  - b) Rechtsgrundlage 17
  - c) Verantwortlichkeit 17
  - d) Zweck(e) 18
  - e) Weitergabe/Empfänger 18
  - f) Ihre Rechte 20
  - g) Dauer der Speicherung der Daten: 24
  - h) Veröffentlichung 24
- II. 2. Was geschieht mit meinen Bioproben? 24
  - a) Verwendung Ihrer Bioproben 25
  - b) Lagerung 25
  - c) Weitergabe/Empfänger 25
  - d) Umgang mit den Bioproben bei Widerruf/vorzeitiger Beendigung der Teilnahme 25

Anlagen 31

Teil I: Informationen zum Ablauf der klinischen Prüfung und zu gesundheitlichen Aspekten

I. 1. Warum und mit welchem Prüfpräparat wird diese Prüfung durchgeführt?

Menschen mit einem Post COVID Syndrom haben über viele Monate hinweg Beschwerden. Diese Beschwerden können sehr verschieden sein, und man weiß noch nicht genau, warum sie entstehen. Es könnte sein, dass das Corona-Virus auch nach der ersten Krankheitsphase im Körper bleibt. Eine andere Möglichkeit ist, dass die Corona-Infektion das Immunsystem so schwächt, dass Viren, die schon vorher im Körper waren, wieder aktiv werden. In beiden Fällen könnte eine Behandlung mit Medikamenten, die Viren bekämpfen, helfen. Bisher wurde noch nicht untersucht, ob solche Medikamente bei der Behandlung des Post COVID Syndroms helfen können. Eine dritte Möglichkeit ist eine nicht aufhörende Überreaktion des Immunsystems auf die Corona-Infektion. Daher könnten auch Medikamente, die das Immunsystem in seiner Aktivität hemmen, hilfreich sein.

Es ist allerdings gezeigt worden, dass das antivirale Medikament IMU-838 die Dauer der akuten Corona-Infektion verkürzt und gleichzeitig die Aktivität des Immunsystems hemmt. Daher könnte IMU-838 auch gegen das Post COVID Syndrom wirken. Ob dies der Fall ist, soll in dieser Studie untersucht werden. Bei IMU-838 handelt es sich um eine Tablette, die den Wirkstoff Vidofludimus-Calcium (VidoCa) enthält.

I. 2. Erhalte ich das Prüfpräparat auf jeden Fall?

IMU-838 ist ein Arzneimittel in klinischer Erprobung. Das heißt, es ist für die Behandlung des Post COVID Syndroms noch nicht zugelassen. Es wurde bisher bei ungefähr 1400 Personen, davon 200 mit akuter Corona-Infektion geprüft. Bei Patienten mit einem Post COVID Syndrom wurde es noch nicht getestet.

Nicht alle Teilnehmer erhalten das Prüfpräparat IMU-838. Im Rahmen dieser klinischen Prüfung wird IMU-838 mit einem Placebo verglichen. Ein Placebo ist eine Tablette, die genauso aussieht wie das Prüfpräparat, aber keinen Wirkstoff enthält. Der Vergleich mit dem Placebo dient dazu, die Wirkungen und Nebenwirkungen von IMU-838 besser beurteilen zu können.

Ob Sie ein Placebo oder das Prüfpräparat IMU-838 erhalten, entscheidet ein Zufallsverfahren. Dieses Verfahren nennt man Randomisierung. Es ist vergleichbar mit dem Werfen einer Münze. Die Wahrscheinlichkeit, Placebo oder IMU-838 zu bekommen, ist jeweils gleich groß. Sie beträgt also jeweils 50%.

Um objektive Studiendaten zu erhalten, ist es notwendig, dass weder Sie noch Ihr Prüfarzt wissen, ob Sie das Prüfpräparat oder das Placebo einnehmen. Dieses Vorgehen nennt man

„doppelblind“. Sollte es aus Sicherheitsgründen notwendig sein, kann sofort festgestellt werden, welche Behandlung Sie im Rahmen der Studie erhalten haben.

Wenn Sie an der Studie teilnehmen, werden Sie die Studientabletten über einen Zeitraum von 56 Tagen einnehmen.

I. 3. Wie ist der Ablauf der Studie und was ist bei der Teilnahme zu beachten?

Bei der geplanten Studie handelt es sich um eine sogenannte "Plattformstudie". Wir möchten kurz erklären, was das bedeutet.

Bei Plattformstudien wird einerseits geprüft, ob ein bestimmtes Medikament wirkt. Andererseits eröffnen sie die Möglichkeit, diese Wirkung im Verlauf der Studie mit der Wirkung anderer Medikamente oder nicht-medikamentöser Behandlungen zu vergleichen. Das bedeutet, dass während der Studie verschiedene Behandlungen erprobt und manchmal auch abgebrochen werden können. Der genaue Verlauf der Studie steht also nicht von Anfang an fest.

Wir werden in regelmäßigen Abständen überprüfen, ob IMU-838 gut wirkt oder ob andere Behandlungen erfolgversprechend sind. Das kann dazu führen, dass wir den Studienplan ändern. In diesem Fall werden wir Sie informieren und fragen, ob Sie weiterhin an der Studie teilnehmen möchten.

Wenn Sie sich jetzt für eine Teilnahme entscheiden, sieht der Ablauf wie folgt aus:

Für die Teilnahme an der Studie sollten Sie ein Smartphone oder Tablett besitzen und bedienen können. In Einzelfällen können wir aber auch ein solches Gerät zur Verfügung stellen. Bei Aufnahme in diese klinische Prüfung werden Sie zu Ihren Vorerkrankungen und Ihrem aktuellen Gesundheitsstatus befragt und Sie werden vom Arzt untersucht. Außerdem absolvieren Sie zwei kurze Tests, um Ihre körperliche und geistige Leistungsfähigkeit festzustellen und füllen zwei kurze Fragebögen aus. Zudem benötigen wir von Ihnen ca. 20 ml Blut für Laboruntersuchungen. Wenn entsprechende Laboruntersuchungen bereits aus der klinischen Versorgung vorliegen, können wir auf Teile dieser Untersuchungen verzichten und die vorliegenden Daten verwenden.

Die Möglichkeit einer weiteren Teilnahme an dieser klinischen Prüfung wird von den Ergebnissen dieser Voruntersuchung abhängen.

Nach der Aufnahme in die klinische Prüfung wird diese für jeden Teilnehmer voraussichtlich drei Monate dauern. Sie erhalten von uns zusätzlich zu dieser Information einen Terminplan zur Orientierung (Anlage 1).

Tag -7
Sie kommen zur Untersuchung ins Prüfzentrum. Die Untersuchung an diesem Tag wird etwa 6 Stunden dauern. Wir führen mit Ihnen einen Test durch, um Ihre körperliche und geistige Leistungsfähigkeit festzustellen. Außerdem benötigen wir von Ihnen eine Stuhlprobe und nehmen Ihnen etwa 50 ml Blut ab. Darüber hinaus werden wir Sie bitten, 8 Fragebögen

auszufüllen. Dafür wird auf Ihrem Handy eine App installiert und Ihnen gezeigt, wie diese benutzt wird. Eine schriftliche Gebrauchsanweisung finden Sie in Anlage 2.

Bevor Sie mit der Einnahme der Tabletten beginnen, werden Sie 7 Tage lang einen Ohrsensor und eine Armbanduhr (Smartwatch) tragen. Diese messen Ihre Körpertemperatur, Herzfrequenz, Atemfrequenz, den Sauerstoffgehalt Ihres Blutes und wie viel Sie sich bewegen. Daraus können wir ableiten, wie gut Ihr Herz-Kreislauf-System funktioniert und wie aktiv Sie sind. Wir weisen Sie in die Handhabung des Ohrsensors und der Armbanduhr ein. Eine schriftliche Gebrauchsanweisung finden Sie in Anlage 2.

Bei den ersten 100 Teilnehmern wird, wenn diese an dieser Untersuchung Interesse haben, innerhalb dieser 7 Tage auch eine Kernspintomographie des Kopfes durchgeführt. Zusätzliche Informationen hierzu finden Sie weiter unten.

Tag 1 (erster Tag der Tabletteneinnahme)

Nachdem Sie 7 Tage den Ohrsensor und die Armbanduhr getragen haben, kommen Sie erneut ins Prüfzentrum. Dort erhalten Sie die Tabletten (IMU-838 oder Placebo) für die ersten 28 Tage. Sie erhalten zwei verschiedene Flaschen. In der kleineren befinden sich die Tabletten für die ersten 7 Tage. Diese haben eine niedrigere Dosierung von 22,5 mg VidoCa (oder Placebo); zum Einschleichen der Medikation. Wenn die kleinere Flasche nach 7 Tagen aufgebraucht ist, beginnen Sie mit den Tabletten aus der größeren Flasche. In dieser Flasche befinden sich 30 Tabletten mit 45 mg VidoCa (oder Placebo).

Tag 14

Wir erkundigen uns bei Ihnen erneut per Videotelefonat oder telefonisch nach Ihrem Befinden.

Tag 21

An diesem Tag starten Sie zu Hause die Messung mit dem Ohrsensor und der Armbanduhr und führen diese wieder über 7 Tage durch.

Tag 28

Sie kommen an diesem Tag zu uns in das Prüfzentrum. Die Untersuchung an diesem Tag dauert etwa 2 Stunden.

Wir benötigen erneut eine Stuhlprobe und nehmen Ihnen etwa 60 ml Blut ab. Wir führen erneut mit Ihnen drei kurze Tests durch, um Ihre körperliche und geistige Leistungsfähigkeit festzustellen. Außerdem werden Sie 8 Fragebögen über Ihre App beantworten. Sie erhalten die Tabletten für die nächsten 28 Tage von uns.

Tag 49

An diesem Tag starten Sie erneut zu Hause die Messung mit dem Ohrsensor und der Armbanduhr und führen diese wieder über 7 Tage durch. Die Geräte bringen Sie an Tag 56 mit in das Prüfzentrum.

Tag 56 (letzter Tag der Tabletteneinnahme)
Nach Einnahme der letzten Tablette kommen Sie zu uns in das Prüfzentrum. Die Untersuchung an diesem Tag dauert etwa 2 Stunden.
Wir werden die gleichen Untersuchungen wie an Tag 28 durchführen (Stuhlprobe, etwa 60 ml Blut, 8 Fragebögen per App, körperliche und geistige Leistungstests). Zusätzlich übergeben Sie uns den Ohrsensor und die Armbanduhr.

Tag 84
Wir erkundigen uns bei Ihnen telefonisch oder per Videotelefonat nach Ihrem Befinden.
Zu diesem Zeitpunkt füllen Sie bitte ein letztes Mal die 8 Fragebögen per App aus.

Einnahme der Tabletten – was ist zu beachten?
Sie müssen die Studientabletten ab dem 1. Tag über einen Zeitraum von 56 Tagen einnehmen. Schlucken Sie jeweils 1 Tablette täglich unzerkaut etwa eine halbe Stunde vor dem Frühstück mit einem großen Glas Wasser. Insgesamt sollten Sie während der Studienteilnahme ausreichend Flüssigkeit zu sich nehmen, in jedem Fall mindestens 1,5 Liter am Tag.

Bewahren Sie die Tabletten an einem sicheren, dunklen und trockenen Ort bei Zimmertemperatur auf. Sorgen Sie dafür, dass sie für Kinder oder andere Personen nicht erreichbar sind. Die Weitergabe der Studientabletten an Dritte ist untersagt.

Zusätzliche Medikamente (auch rezeptfreie), von denen der Prüfarzt noch nichts weiß, dürfen Sie – außer bei Notfällen – nur nach Rücksprache mit Ihrem Prüfarzt einnehmen. Wenn Sie von anderen Ärzten behandelt werden, müssen Sie diese über Ihre Teilnahme an der klinischen Prüfung informieren. Auch Ihr Prüfarzt muss über jede medizinische Behandlung, die Sie durch einen anderen Arzt während der klinischen Prüfung erhalten, informiert werden. Sie erhalten einen Studienausweis, den Sie auch für den Notfall immer mit sich führen sollten.

Bitte beachten sie, dass IMU-838 die Wirkung anderer Medikamente abschwächen oder verstärken kann. Nehmen Sie daher insbesondere keine Schmerztabletten wie Ibuprofen, Diclofenac oder Mittel gegen Übelkeit, ohne mit Ihrem Arzt zu sprechen.
Zu den Untersuchungen im Prüfzentrum sollten Sie die Packung Ihrer Studienmedikation mitbringen, egal ob sie leer ist oder nicht.

I. 4. Welchen persönlichen Nutzen habe ich von der Teilnahme an der Studie?

Die Teilnahme an dieser Studie kann möglicherweise Ihre Beschwerden lindern oder verkürzen. Für das Prüfpräparat IMU-838 gibt es jedoch noch keinen Nachweis für seine Wirksamkeit bei der Therapie des Post COVID Syndroms. Es ist daher möglich, dass Sie von der Teilnahme an dieser klinischen Prüfung keinen Nutzen haben. Die Ergebnisse der Studie können aber dazu beitragen, die Behandlung des Post COVID Syndroms zukünftig zu verbessern.

I. 5. Welche gesundheitlichen Risiken und Belastungen sind mit der Teilnahme an der Studie verbunden?

Bitte teilen Sie den Mitarbeitern der Prüfzentrum **alle** Beschwerden, Erkrankungen oder Verletzungen mit, die im Verlauf der klinischen Prüfung auftreten. Falls Sie diese als schwerwiegend empfinden, teilen Sie den Mitarbeitern der Prüfzentrum diese bitte umgehend mit, gegebenenfalls telefonisch. Die Kontaktdaten finden Sie vorne auf dieser Information.

a) Risiken durch das Prüfmedikament IMU-838

Bisher wurden bei 1400 Patienten in verschiedenen Studien keine schwerwiegenden Nebenwirkungen festgestellt. Die häufigsten Nebenwirkungen waren Kopfschmerz (10%) und Erkältungssymptome (1-4%). Wurde IMU-838 in der Dosis gegeben, wie es in dieser Studie der Fall ist, dann hatten die Teilnehmer mit IMU-838 ähnlich häufig diese Nebenwirkungen, wie die Teilnehmer, die das Scheinmedikament (Placebo) genommen haben.

Bei der Einnahme von hohen Dosen von IMU-838 – höher als Sie sie für diese Studie einnehmen werden – wurde vereinzelt Blut im Urin gefunden. Sollten Sie Auffälligkeiten beim Wasserlassen bemerken oder neue Schmerzen in der Flankenregion, teilen Sie dies Ihrem Prüfarzt bitte umgehend mit. Er wird das weitere Vorgehen mit Ihnen besprechen. Es ist aber sehr unwahrscheinlich, dass dies eintreten wird.

Wie bei jedem neuen Medikament kann es auch mit IMU-838 Nebenwirkungen geben, die noch nicht bekannt sind. Viele Nebenwirkungen verschwinden, wenn das Studienmedikament abgesetzt wird, aber in einigen Fällen können die Nebenwirkungen schwerwiegend und/oder dauerhaft sein.

Jedes Medikament kann eine allergische Reaktion auslösen. Informieren Sie sofort Ihren Studienarzt, wenn Sie eines der folgenden Symptome bemerken:

- Plötzliches Anschwellen der Lippen, des Gesichts, des Rachens oder der Zunge,
- schwerer Hautausschlag,
- Schluck- oder Atembeschwerden.

Informieren Sie sofort Ihren Prüfarzt, wenn Sie eine allergische Reaktion vermuten.

Klinische Studien mit anderen Arzneimitteln, die einen ähnlichen Wirkmechanismus wie IMU-838 haben, zeigen, dass diese Medikamente die Wirksamkeit von Impfungen nicht beeinträchtigen. Es liegen jedoch keine klinischen Daten über IMU-838 vor. Da Impfungen die Symptome des Post COVID Syndroms beeinflussen können und daher die Auswertbarkeit der Studie beeinflussen können, sollten Sie während des Studienzeitraums keine Impfung erhalten, wenn es sich vermeiden lässt.

Bitte sprechen Sie mit Ihrem Studienarzt, wenn Sie sich trotzdem impfen lassen möchten. Ihr Studienarzt wird Sie über die potenziellen Risiken und Vorteile einer solchen Impfung und Ihrer Teilnahme an dieser Forschungsstudie beraten können.

Weitere Informationen können Sie der angehängten Beschreibung (Anlage 3) aller bisher aufgetretener Nebenwirkungen entnehmen.

b) Risiken durch studienbedingte Maßnahmen

<u>Blutabnahmen</u>
Im Rahmen der Studie werden wir Ihnen einmal etwa 20 ml, einmal etwa 50 ml und zweimal etwa 60 ml Blut abnehmen. 20 ml Blut entsprechen etwa einem Esslöffel. In der Regel ist das Risiko einer Blutentnahme sehr gering. Sie ist jedoch manchmal unangenehm und mit Schmerzen verbunden. Sehr selten kann es zu einer Verletzung und dauerhaften Schädigung eines Nervs oder zu einem größeren Bluterguss an der Einstichstelle kommen.

<u>Magnetresonanztomographie (MRT) des Kopfes</u>
Mit Hilfe von Magnetfeldern werden bei einer MRT-Untersuchung genaue Bilder des Gehirns erzeugt. Sie werden dazu in eine Art Röhre gelegt. Manche Patienten können dadurch während der Untersuchung Unbehagen verspüren, insbesondere, wenn sie unter Platzangst leiden. In solchen Fällen kann es hilfreich sein, vor der Untersuchung beruhigende Maßnahmen zu ergreifen. Außerdem ist die Untersuchung relativ laut. Sie tragen daher Ohrschützer.

<u>Zufallsbefunde beim MRT des Kopfes</u>

Es können sich Auswertungsergebnisse ergeben, die möglicherweise für Ihre Gesundheit wichtig sind (Zufallsbefunde). Diese Zufallsbefunde können im Einzelfall für Ihre Gesundheit von so erheblicher Bedeutung sein, dass ein Arzt eine Kontaktaufnahme als dringend notwendig erachtet. Das ist insbesondere der Fall, wenn sich daraus ein dringender Verdacht auf eine schwerwiegende, bisher möglicherweise nicht erkannte Krankheit ergibt, die behandelt werden könnte.

In diesem Fall werden wir einen Facharzt hinzuziehen und Ihre Untersuchungsergebnisse mit ihm besprechen. Wir werden Sie danach anschreiben und gemeinsam mit dem Facharzt ein Gespräch mit Ihnen führen, um das weitere Vorgehen zu besprechen.

Beachten Sie bitte, dass Sie Gesundheitsinformationen, die Sie durch eine solche Rückmeldung erhalten, unter Umständen bei anderen Stellen (z.B. vor Abschluss einer Kranken- oder Lebensversicherung) angeben müssen. Dadurch könnten Sie Nachteile erleiden. Im Zweifel verzichten Sie auf die MRT-Untersuchung. Eine Studienteilnahme ist dennoch möglich.

<u>Ohrsensor</u>

Wenn Sie an dieser Studie teilnehmen, tragen Sie 3-mal für jeweils 7 Tage einen Ohrsensor. Der Ohrsensor wird wie ein Hörgerät im rechten oder linken Ohr getragen. Das Tragen kann als unangenehm empfunden werden, da der Sensor unter Umständen drücken kann. Wir versuchen, eine möglichst passende Größe für Ihr Ohr zu finden. Ganz selten kann der Sensor eine allergische Reaktion hervorrufen; wenn Sie dies vermuten, kontaktieren Sie uns bitte.

<u>Armbanduhr / Smartwatch</u>

Wenn Sie an dieser Studie teilnehmen, tragen Sie 3-mal für jeweils 7 Tage eine Uhr (Smartwatch). Die Uhr kann am rechten oder linken Handgelenk getragen werden. Das Display und der Messsensor geben etwas Licht ab, was von einigen Menschen nachts als störend empfunden werden kann. Ganz selten kann das Armband eine allergische Reaktion hervorrufen; wenn Sie dies vermuten, kontaktieren Sie uns bitte.

<u>Fragebögen</u>

Die in dieser Studie verwendeten Fragebögen können unangenehme Gefühle auslösen. Aus wissenschaftlicher Sicht ist es wünschenswert, von allen Studienteilnehmern vollständig ausgefüllte Fragebögen zu erhalten. Sie sind jedoch nicht verpflichtet, Fragen zu beantworten, die Ihnen unangenehm sind.

I. 6. Welche anderen Behandlungsmöglichkeiten gibt es außerhalb der Studie?

Bisher gibt es keine Therapien, die die Ursache des Post COVID Syndroms gezielt behandeln. Vielmehr wird versucht, die individuell vorliegenden Symptome mit bereits zugelassenen Medikamenten zu lindern. Diese Behandlung ist zurzeit die Standardbehandlung für Ihre Erkrankung. Im Vergleich zur Studienteilnahme kann diese sog. symptomatische Therapie die Ursachen Ihrer Erkrankung nicht gezielt behandeln. Es ist also nicht damit zu rechnen, dass nach Absetzen einer symptomatischen Therapie eine längerfristige Besserung eintritt.

Über weitere Einzelheiten kann Sie der Prüfarzt informieren.

I. 7. Wer darf an dieser klinischen Prüfung nicht teilnehmen?

An dieser klinischen Prüfung dürfen Sie nicht teilnehmen, wenn Sie gleichzeitig an einer anderen Arzneimittelstudie teilnehmen oder in den vergangenen 6 Monaten teilgenommen haben.

Es ist bislang nicht geklärt, ob es zu einer Schädigung des Ungeborenen kommt, wenn das Prüfpräparat IMU-838 während der Schwangerschaft eingenommen wird. Studien an Ratten mit sehr hohen Dosen des Prüfpräparats ergaben jedoch unerwünschte Wirkungen bei den ungeborenen Tieren. Daher dürfen **schwangere** oder **stillende Frauen nicht** an dieser Studie teilnehmen. Zu Beginn der klinischen Prüfung müssen sich deshalb alle Frauen einem Schwangerschaftstest unterziehen. Davon ausgenommen sind Frauen, die nicht mehr schwanger werden können.

Wenn Sie aktuell planen, schwanger zu werden oder Eizellen zu spenden, dürfen Sie einer Teilnahme nicht zustimmen. Für männliche Teilnehmer gilt: sollten Sie planen, während des Zeitraums dieser Studie ein Kind zu zeugen oder Sperma zu spenden, dürfen Sie der Teilnahme nicht zustimmen.

Maßnahmen zur Schwangerschaftsverhütung:
Im Falle einer Teilnahme an dieser klinischen Prüfung müssen Sie eine Schwangerschaft zuverlässig verhüten. Sie müssen entweder mindestens eine hochwirksame Verhütungsmethode anwenden (z. B. eine Spirale oder ein Implantat) oder Sie müssen zwei wirksame Verhütungsmethoden, die auf unterschiedliche Weise funktionieren, korrekt anwenden (z. B. eine hormonelle Verhütungspille in Kombination mit einem Kondom). Dies gilt ab einem Monat vor Beginn der Teilnahme, während der Studie sowie bis 30 Tage nach der letzten Dosis des Studienmedikaments (Tag 86).

Wenn Sie während dieser Studie schwanger werden, müssen Sie dies sofort dem Prüfarzt mitteilen. Sie müssen das Studienmedikament sofort absetzen und der Studienarzt wird mit Ihnen und Ihrem Partner/Ihrer Partnerin die Möglichkeiten zum Umgang mit der Schwangerschaft besprechen. Sie werden während der ganzen Schwangerschaft und bis 30 Tage nach der Entbindung überwacht und der Prüfarzt wird Sie außerdem bitten, weitere Informationen über den Verlauf Ihrer Schwangerschaft und die Gesundheit Ihres Babys einholen zu dürfen. Bis zur Geburt ist dazu Ihre Einwilligung ausreichend, ab dem Zeitpunkt der Geburt ist die Einwilligung beider Erziehungsberechtigter notwendig. Sie bekommen im Falle einer Schwangerschaft die notwendigen Dokumente ausgehändigt.

Hinweise für männliche Patienten mit einer Partnerin im gebärfähigen Alter:
Sie müssen sich verpflichten, ab der Zustimmung zur Teilnahme an der Studie und bis 30 Tage nach der letzten Dosis des Studienmedikaments (Tag 86) kein Kind zu zeugen.

Hinweise im Falle einer Schwangerschaft:
Wenn eine Teilnehmerin oder eine Partnerin eines Teilnehmers trotzdem schwanger wird, kann das Studienmedikament unvorhersehbare Risiken für das ungeborene Kind bergen. Wir möchten in dem Fall die Gesundheit des Kindes bis zum Ende der Schwangerschaft überwachen. Dafür wird es dann eine gesonderte Information- und Einwilligungserklärung geben.

I. 8. Entstehen für mich Kosten durch die Teilnahme an der klinischen Prüfung? Erhalte ich eine Aufwandsentschädigung?

Für die Teilnehmer an dieser klinischen Prüfung entstehen keine zusätzlichen Kosten.

Es gibt keine Aufwandsentschädigung für die Teilnahme an dieser klinischen Prüfung.

I. 9. Bin ich während der klinischen Prüfung versichert?

Bei der klinischen Prüfung eines Arzneimittels müssen alle Studienteilnehmer gemäß dem Arzneimittelgesetz versichert sein. Der Umfang des Versicherungsschutzes ergibt sich aus den Versicherungsunterlagen, die Sie ausgehändigt bekommen.

Wenn Sie vermuten, dass durch die Teilnahme an der klinischen Prüfung Ihre Gesundheit geschädigt oder vorher bestehende Leiden verstärkt wurden, müssen Sie dies unverzüglich dem

Versicherer

Kontaktdaten des Versicherers:

Chubb European Group SE
Direktion für Deutschland
Baseler Straße 10
60329 Frankfurt am Main
Telefon: +49 69 75613 0
Fax: +49 69 746193

E-Mail: info.de@chubb.com

Versicherungsscheinnummer:

DELSCA48846

direkt anzeigen, um Ihren Versicherungsschutz nicht zu gefährden. Lassen Sie sich dabei gegebenenfalls durch Ihren Prüfarzt unterstützen. Sofern Ihr Prüfarzt Sie dabei unterstützt, erhalten Sie eine Kopie der Meldung. Sofern Sie Ihre Anzeige direkt an den Versicherer richten, informieren Sie bitte zusätzlich Ihren Prüfarzt.

Bei der Aufklärung der Ursache oder des Umfangs eines Schadens müssen Sie mitwirken und alles unternehmen, um weiteren Schaden abzuwenden.

Während der Dauer der klinischen Prüfung dürfen Sie sich einer anderen medizinischen Behandlung – außer in Notfällen – nur nach vorheriger Rücksprache mit dem Prüfarzt unterziehen. Von einer erfolgten Notfallbehandlung müssen Sie den Prüfarzt unverzüglich unterrichten.

Sie erhalten ein Exemplar der Versicherungsbestätigung einschließlich der Versicherungsbedingungen. Wir weisen Sie insbesondere auf Kapitel 1.4 zum Ausschluss von Versicherungsleistungen und Kapitel 3.1 zum Umfang der Versicherungsleistung hin. Bitte beachten Sie außerdem die Kapitel 4.3 und 4.4.2 zu den Obliegenheiten/Verpflichtungen (das sind Pflichten, die Sie im eigenen Interesse beachten müssen, um den Versicherungsschutz zu erhalten).

Wir weisen Sie ferner darauf hin, dass für die Teilnehmer auf dem Weg von und zum Prüfzentrum eine Unfallversicherung besteht. Der Umfang des Versicherungsschutzes ergibt sich aus den Versicherungsunterlagen, die Sie ausgehändigt bekommen. Sie erhalten ein Exemplar der Versicherungsbestätigung einschließlich der Versicherungsbedingungen. Im Falle eines Unfalls auf dem Weg zum oder vom Prüfzentrum wenden Sie sich bitte unverzüglich an den

Versicherer:

Chubb European Group SE
Direktion für Deutschland

Baseler Straße 10
60329 Frankfurt am Main
Telefon: +49 69 75613 0
Fax: +49 69 746193

E-Mail: info.de@chubb.com

Versicherungsscheinnummer:

DELSCA48846

I. 10. Werden mir neue Erkenntnisse zu der klinischen Prüfung mitgeteilt?

Sie werden während der Teilnahme über neue Erkenntnisse in Bezug auf diese klinische Prüfung informiert, die für Ihre Bereitschaft zur weiteren Teilnahme wesentlich sein können.

Spätestens ein Jahr nach Beendigung der gesamten klinischen Prüfung müssen zusammenfassende Ergebnisse in der europäischen Datenbank (www.clinicaltrialsregister.eu) – auch in laienverständlicher Form – bereitgestellt werden. Dies kann von Ihnen unter der oben angegebenen EU trial Nummer eingesehen werden. Sie können sich zur Information über die Studienergebnisse auch gerne an Ihren Prüfarzt wenden.

I. 11. Wer entscheidet, ob ich aus der klinischen Prüfung ausscheide?

Sie können jederzeit, auch ohne Angabe von Gründen, Ihre Teilnahme beenden, ohne dass Ihnen dadurch Nachteile in Ihrer medizinischen Versorgung entstehen. Sie können in diesem Fall diejenige Therapie erhalten, die man Ihnen außerhalb der Studie empfohlen hätte.

Es ist auch möglich, dass der Prüfarzt oder der Sponsor entscheidet, Ihre Teilnahme an der klinischen Prüfung vorzeitig zu beenden, ohne dass Sie auf die Entscheidung Einfluss haben.

Die Gründe hierfür können z.B. sein:

- Ihre weitere Teilnahme an der klinischen Prüfung ist ärztlich nicht mehr vertretbar;
- es wird die gesamte klinische Prüfung abgebrochen.

Wenn Sie die klinische Prüfung vorzeitig beenden, ist es für Ihre eigene Sicherheit wichtig, dass eine Abschlussuntersuchung durchgeführt wird.

Der Prüfarzt wird mit Ihnen besprechen, wie und wo die weitere Behandlung stattfindet.

I. 12. An wen wende ich mich bei weiteren Fragen?

Beratungsgespräche an dem Prüfzentrum:

Sie haben stets die Gelegenheit zu weiteren Beratungsgesprächen mit dem vorne auf der Information genannten oder einem anderen Prüfarzt.

Kontaktstelle:

Es existiert außerdem eine Kontaktstelle bei der zuständigen Bundesoberbehörde. Teilnehmer an klinischen Prüfungen, ihre gesetzlichen Vertreter oder Bevollmächtigte können sich an diese Kontaktstelle wenden:

Bundesinstitut für Arzneimittel und Medizinprodukte
Fachgruppe Klinische Prüfungen
Kurt-Georg-Kiesinger-Allee 3
53175 Bonn
Telefon: 0228 / 207-4318   Fax: 0228 / 207-4355
E-Mail: ct@bfarm.de

Teil II: Informationen zum Datenschutz und zu den Bioproben

II. 1. Was geschieht mit den über mich erhobenen Daten?

a) Allgemeine Informationen

Während der klinischen Prüfung werden persönliche Informationen (etwa Alter, Geschlecht, etc.) und medizinische Befunde (Gesundheitsdaten) von Ihnen erhoben und im Prüfzentrum in Ihrer persönlichen Akte niedergeschrieben oder elektronisch gespeichert. Eine unabhängige Treuhandstelle (Unabhängige Treuhandstelle der Universitätsmedizin Greifswald, Ellernholzstr. 1-2, 17475 Greifswald; https://www.ths-greifswald.de/kontakt/) speichert Ihre identifizierenden Daten und einen Scan Ihrer Einwilligungserklärung. Die Treuhandstelle sendet einen Pseudonymisierungscode an die datenhaltenden Stellen. Diese speichern und verarbeiten Daten nur in pseudonymisierter Form.

Pseudonymisiert bedeutet, dass keine Angaben, mit denen Sie direkt identifiziert werden können (z.B. Namen, Kontaktinformationen, Geburtsdatum etc.), verwendet werden, sondern nur ein Nummern- und/oder Buchstabencode. Dem jeweiligen Prüfzentrum steht eine Pseudonymisierungsliste zur Verfügung, die dort gegen unbefugten Zugriff geschützt verbleibt. Das ist notwendig, damit Ihnen diese personenbezogenen Daten, falls erforderlich, wieder zugeordnet werden können (Entschlüsselung). Eine solche Entschlüsselung geschieht nur, wenn es im Rahmen der Studie zum Schutz Ihrer Gesundheit notwendig ist. In diesem Rahmen ist eine Entschlüsselung rechtlich zulässig. Es lässt sich allerdings niemals völlig ausschließen, dass auch ohne diese Liste zur Entschlüsselung Rückschlüsse auf Ihre Person gezogen werden könnten.

Die pseudonymisierten Daten werden insbesondere an den Sponsor dieser klinischen Prüfung weitergegeben und dort gespeichert (siehe II. 1. Buchst. e) Weitergabe/Empfänger).

b) Rechtsgrundlage

Rechtsgrundlage für die Datenverarbeitung ist Ihre informierte Einwilligung gemäß Art. 6 Abs. 1 Buchst. a und Art. 9 Abs. 2 Buchst. a der EU Datenschutzgrundverordnung (DSGVO) sowie § 40b Abs. 6 Arzneimittelgesetz (AMG) und Art. 9 Abs. 2 Buchstabe j DSGVO.

Die Bereitstellung Ihrer personenbezogenen Daten ist freiwillig. Ohne Ihre ausdrückliche Einwilligung in die Verarbeitung Ihrer Daten können Sie allerdings nicht an dieser klinischen Prüfung teilnehmen.

c) Verantwortlichkeit

Verantwortlich im Sinne des Datenschutzrechts ist der Sponsor der klinischen Prüfung, Goethe-Universität Frankfurt, ebenso wie das Prüfzentrum. Das Prüfzentrum bleibt davon unabhängig für die Behandlungsdaten verantwortlich (unkodierte Patientendaten).

d) Zweck(e)

Mit Hilfe der erhobenen Daten soll die Unbedenklichkeit oder Wirksamkeit von IMU-838 bei Menschen, die am Post COVID Syndrom leiden, klinisch untersucht werden.

Zudem werden die erhobenen Daten auch bei den notwendigen Zulassungsverfahren für das zu untersuchende Arzneimittel verwendet.

Wir möchten Ihnen die Möglichkeit geben, Ihre Daten und Bioproben für spätere medizinische Forschungsprojekte zur Verfügung zu stellen. Eine Entscheidung dafür oder dagegen ist freiwillig, jederzeit widerrufbar, und hat keine Auswirkung auf Ihre Teilnahme an dieser Studie. Bitte beachten Sie dazu die Patienteninformation „Optionale zusätzliche Sammlung von Bioproben und Nutzung von Daten im Rahmen der klinischen Arzneimittelprüfung: Randomized adaptive assessment of post COVID syndrome treatments_Reducing Inflammatory Activity in Patients with post COVID Syndrome“.

e) Weitergabe/Empfänger

Die für die klinische Prüfung wichtigen Daten und Bioproben werden zusätzlich in pseudonymisierter Form verarbeitet und gegebenenfalls weitergegeben.

Die erhobenen Daten werden, soweit erforderlich, pseudonymisiert weitergegeben an:

1) den Sponsor, Goethe-Universität Frankfurt, und von diesem beauftragte Stellen zum Zweck der Durchführung und wissenschaftlichen Auswertung,

Institut für Digitale Medizin
Universitätsklinikum Gießen und Marburg GmbH (UKGM), Philipps-Universität Marburg
Baldingerstraße
35042 Marburg

Klinikum rechts der Isar, Technische Universität München
Innere Medizin I, Arbeitsgruppe Biosignalanalyse
Ismaninger Str. 22
81675 München

Universitätsklinikum Frankfurt, Institut für Medizinische Virologie
Paul-Ehrlich-Straße 40, 60596 Frankfurt am Main

Charité-Universitätsmedizin Berlin,
Berlin Institute of Health at Charité (BIH), Translationale Immunologie
Campus Virchow-Klinikum

Augustenburger Platz 1
D-13353 Berlin

Unabhängige Treuhandstelle der Universitätsmedizin Greifswald K.d.ö.R.
Ellernholzstr. 1-2
17475 Greifswald

Hannover Unified Biobank (HUB)
Medizinische Hochschule Hannover (MHH)
Feodor-Lynen-Str.15
30625 Hannover

Zentrale Biobank der Universität Bielefeld
Medizinische Fakultät OWL
Universität Bielefeld
Universitätsstraße 25
33615 Bielefeld

Charité – Universitätsmedizin Berlin,
Institut für kardiovaskuläre Computer-assistierte Medizin (ICM)
Campus Virchow-Klinikum
Augustenburger Platz 1
13353 Berlin

Universitätsmedizin Greifswald
Institut für Klinische Chemie und Laboratoriumsmedizin
Ferdinand-Sauerbruch-Straße
17475 Greifswald

Medizinische Informatik Göttingen
Institut für Medizinische Informatik
Universitätsmedizin Göttingen der Georg-August-Universität
Robert-Koch-Str. 40, 37075 Göttingen

Studienzentrum Universitätsmedizin Göttingen der Georg-August-Universität
Von-Bar-Straße 2/4, 37075 Göttingen

2) im Falle unerwünschter Ereignisse: an den Sponsor,

- sowie von diesem ggf. an die zuständigen Behörden der Mitgliedstaaten der Europäischen Union oder des Abkommens über den Europäischen Wirtschaftsraum, in deren Hoheitsgebiet die o.g. klinische Prüfung durchgeführt wird

- oder an die für die Arzneimittelsicherheit eingerichtete Europäische Datenbank (EudraVigilance), auf die die zuständigen Überwachungsbehörden in der gesamten Europäischen Union und dem Europäischen Wirtschaftsraum Zugriff haben,

3) im Fall eines Antrags auf Zulassung als Arzneimittel an den Antragsteller und die für die Zulassung zuständige Behörde.

Die von Ihnen im Rahmen der oben genannten klinischen Studie erhobenen und gespeicherten Daten (auch die originalen Klardaten) können soweit erforderlich und gesetzlich erlaubt, durch die zuständige Überwachungsbehörde im Rahmen von Inspektionen oder durch Beauftragte des Sponsors (s.g. Auditoren oder Monitore) zur Überprüfung der ordnungsgemäßen Durchführung der klinischen Prüfung im Prüfzentrum eingesehen werden. Diese Personen sind zur Vertraulichkeit verpflichtet, eine Weitergabe der erhobenen Daten erfolgt in diesem Zusammenhang nicht.

Für diese Studie hat die Immunic AG die Prüfsubstanz bereitgestellt. Für Forschungszwecke gibt es die Möglichkeit für die Immunic AG ihre Daten zu kaufen. Dabei findet eine Übermittlung ihrer Daten in verschlüsselter Form an die Immunic AG oder von ihr beauftragte Unternehmen in den USA statt. Die Übermittlung von Daten in die USA erfolgt gemäß einem Angemessenheitsbeschluss der EU-Kommission. Dieser Beschluss besagt, dass die USA ein angemessenes Schutzniveau bieten. Die entsprechenden Beschlüsse der Kommission sind im Amtsblatt der EU sowie auf der Website der Kommission veröffentlicht und können unter folgendem Link eingesehen werden:

https://commission.europa.eu/document/fa09cbad-dd7d-4684-ae60-be03fcb0fddf_en

Die Daten werden nur an solche beauftragten Dritten übermittelt, die sich freiwillig gemäß dem o.g. Beschluss in den USA registriert haben oder mit denen Standarddatenschutzklauseln der Kommission oder einer Aufsichtsbehörde gemäß Art. 46 Abs. 2 lit. c und d DSGVO vertraglich vereinbart sind.

Bitte beachten Sie, dass Ihre Armbanduhr/Smartwatch ausschließlich mit der SaniQ App verbunden werden sollte. Bei der Verbindung mit anderen Apps, wie etwa solchen des Herstellers der Uhr (z. B. Garmin Connect™) kann der Schutz Ihrer Daten nicht gewährleistet werden.

f) Ihre Rechte

Sie haben grundsätzlich folgende Rechte bezüglich Ihrer personenbezogenen Daten, sofern dies nicht aufgrund einer zwischenzeitlich vorgenommenen Löschung der identifizierenden Merkmale zur Entschlüsselung technisch oder anderweitig gesetzlich unmöglich ist:

Recht auf Widerruf Ihrer Einwilligung

So wie die Einwilligung zur Teilnahme an der klinischen Prüfung können Sie auch Ihre Einwilligung zur Verarbeitung der erhobenen Daten jederzeit widerrufen. Gemäß § 40b Abs. 6 Nr. 2 AMG dürfen im Falle eines Widerrufs Ihre gespeicherten Daten jedoch weiterverwendet werden, soweit dies erforderlich ist, um

1) die Wirkungen des zu prüfenden Arzneimittels festzustellen,

2) sicherzustellen, dass Ihre schutzwürdigen Interessen nicht beeinträchtigt werden,

3) der Pflicht zur Vorlage vollständiger Zulassungsunterlagen zu genügen.

Im Falle eines Widerrufs Ihrer Einwilligung werden die verantwortlichen Stellen unverzüglich prüfen, inwieweit die gespeicherten Daten noch erforderlich sind. Nicht mehr benötige Daten werden unverzüglich gelöscht, sofern nicht gesetzliche Dokumentations- und Meldepflichten entgegenstehen. Die bis zum Widerruf erfolgte Datenverarbeitung bleibt jedoch rechtmäßig.

Bei Widerruf der Studienteilnahme werden Sie gefragt, ob Sie noch einmal zum eigentlichen Ende des Teilnahmezeitraumes angerufen werden dürfen, um Ihr aktuelles Befinden zu erfragen.

Sie haben weiterhin folgende Rechte

Recht auf Auskunft (inkl. unentgeltlicher Überlassung einer Kopie) über Ihre personenbezogenen Daten, die im Rahmen der klinischen Prüfung erhoben, verarbeitet oder ggf. an Dritte übermittelt werden.

Recht auf Datenübertragung der zu Ihrer Person erhobenen Daten an Sie oder eine bestimmte Stelle.

Recht auf Berichtigung unrichtiger personenbezogener Daten, auf Einschränkung der Verarbeitung und auf Widerspruch gegen die Nutzung der Daten.

Mögliche Einschränkungen Ihrer Rechte

Da die Daten im Rahmen einer klinischen Prüfung eines Arzneimittels gemäß den Bestimmungen des Arzneimittelgesetzes verwendet werden, können die oben genannten Rechte unter Umständen nach Prüfung des Einzelfalls eingeschränkt werden (insbesondere nach Art. 17 Abs. 3 Buchst. d und Art. 89 DSGVO). Dies gilt insbesondere, wenn der Anwendung eines dieser Rechte vertragliche, gesetzlichen und/oder behördlichen Dokumentations- und Meldepflichten entgegenstehen oder die Durchführung der Klinischen Prüfung hierdurch unmöglich gemacht oder ernsthaft beeinträchtigt würde.

Wahrnehmung Ihrer Rechte

Wollen Sie von einem oder mehreren der genannten Rechte Gebrauch machen, kontaktieren Sie bitte Ihren Prüfarzt. Bei Anliegen zur Datenverarbeitung und zur Einhaltung der

datenschutzrechtlichen Anforderungen können Sie sich auch an folgende Datenschutzbeauftragte wenden:

Datenschutzbeauftragter der Prüfzentrum:

Datenschutzbeauftragter der Einrichtung:

XXX
Kontaktdaten des zuständigen Datenschutzbeauftragten
Bezeichnung, Postanschrift, Telefonnummer und E-Mailadresse

Datenschutzbeauftragter des Sponsors:

Datenschutzbeauftragter der Goethe-Universität Frankfurt
Robert-Mayer-Straße 11-15
60325 Frankfurt am Main
Tel.: 069/798-28351

E-Mail: dsb@uni-frankfurt.de

Sie haben grundsätzlich auch das Recht jederzeit den Sponsor selbst zu kontaktieren. Bitte wenden Sie sich jedoch im Regelfall an den Prüfer bzw. den Datenschutzbeauftragten in Ihrem Prüfzentrum, da aufgrund der Pseudonymisierung nur hier Ihre Identität bekannt ist und damit sinnvollerweise weitere Schritte unternommen werden können, bzw. eine unbeabsichtigte Identifikation Ihrer Person durch den Sponsor vermieden werden kann. Die Unabhängige Treuhandstelle steht Ihnen bezüglich Ihrer Betroffenenrechte auch nach Beendigung der Studie langfristig als Ansprechpartner zur Verfügung.

Sie haben außerdem ein Beschwerderecht bei einer Datenschutzaufsichtsbehörde. Sollten Sie Bedenken hinsichtlich des Umgangs mit Ihren personenbezogenen Daten haben, können Sie sich an folgende Stellen wenden:

Datenschutzaufsichtsbehörde des Bundeslandes, in dem Ihr Prüfzentrum liegt [wird pro Zentrum angepasst]

Der Landesbeauftragte für den Datenschutz und die Informationsfreiheit Baden-Württemberg
Postfach 10 29 32
70025 Stuttgart
oder
Lautenschlagerstraße 20
70173 Stuttgart
Telefon: 07 11/61 55 41-0
E-Mail: poststelle@lfdi.bwl.de

Der Bayerische Landesbeauftragte für den Datenschutz
Postfach 22 12 19
80502 München
Telefon: 089/21 26 72-0
E-Mail: poststelle@datenschutz-bayern.de

Berliner Beauftragte für Datenschutz und Informationsfreiheit
Alt-Moabit 59-61
10555 Berlin
Telefon: 030/138 89-0
E-Mail: mailbox@datenschutz-berlin.de

Der Hessische Beauftragte für Datenschutz und Informationsfreiheit
Postfach 31 63
65021 Wiesbaden
Gustav-Stresemann-Ring 1
65189 Wiesbaden
Telefon: 06 11/140 80
E-Mail: poststelle@datenschutz.hessen.de

Der Landesbeauftragte für Datenschutz und Informationsfreiheit Mecklenburg-Vorpommern
Schloss Schwerin
Lennéstraße 1
19053 Schwerin
Telefon: 0385/59494-0
E-Mail: info@datenschutz-mv.de

Die Landesbeauftragte für den Datenschutz Niedersachsen
Prinzenstraße 5
30159 Hannover
Telefon: 05 11/120-45 00
E-Mail: poststelle@lfd.niedersachsen.de

Landesbeauftragte für Datenschutz und Informationsfreiheit Nordrhein-Westfalen
Postfach 20 04 44
40102 Düsseldorf
oder
Kavalleriestraße 2-4
40213 Düsseldorf
Telefon: 02 11/384 24-0
E-Mail: poststelle@ldi.nrw.de

Sächsische Datenschutz- und Transparenzbeauftragte
Postfach 11 01 32
01330 Dresden
oder
Devrientstraße 5
01067 Dresden
Telefon: 03 51 / 85471-101
E-Mail : saechsdsb@slt.sachsen.de

Unabhängiges Landeszentrum für Datenschutz Schleswig-Holstein
Postfach 71 16

24171 Kiel
oder
Holstenstraße 98
24103 Kiel
Telefon: 04 31/988-12 00
E-Mail: mail@datenschutzzentrum.de

Für den Sponsor bzw. dessen Vertreter innerhalb der EU zuständige Datenschutzaufsichtsbehörde:

Der Hessische Beauftragte für Datenschutz und Informationsfreiheit
Postfach 31 63
65021 Wiesbaden
Gustav-Stresemann-Ring 1
65189 Wiesbaden
Telefon: 06 11/140 80

E-Mail: poststelle@datenschutz.hessen.de

Eine Liste aller in Deutschland und der Europäischen Union zuständigen Datenschutzaufsichtsbehörden finden Sie unter: https://www.bfdi.bund.de/DE/Infothek/Anschriften_Links/anschriften_links-node.html

g) Dauer der Speicherung der Daten:

Die erhobenen Daten werden von dem Prüfzentrum und dem Sponsor nach Beendigung oder Abbruch der klinischen Prüfung für 25 Jahre gespeichert. Danach werden die personenbezogenen Daten gelöscht, sofern Sie nicht der weiteren Verwendung Ihrer Daten zu Forschungszwecken zugestimmt hatten.

h) Veröffentlichung

Wissenschaftliche Veröffentlichungen von Ergebnissen dieser klinischen Prüfung erfolgen in einer Form, die keine direkten Rückschlüsse auf Ihre Person zulässt.

II. 2. Was geschieht mit meinen Bioproben?

Während dieser klinischen Prüfung werden Ihnen Bioproben (Blut, Stuhlproben) entnommen; die Proben werden ebenso wie die Daten in pseudonymisierter Form aufbewahrt (siehe zur Pseudonymisierung oben II. 1. Buchst. a)).

Die Pseudonymisierung Ihrer Bioproben bietet allerdings nicht zwangsläufig den gleichen Schutz wie die Pseudonymisierung der erhobenen Daten, da Bioproben immer Informationen zu Ihrer Erbsubstanz beinhalten, die eine Identifizierung ermöglichen könnte.

Da somit aus Ihren Bioproben Informationen gewonnen werden können, gelten die oben unter II. 1. genannten datenschutzrechtlichen Aspekte entsprechend auch für Ihre Bioproben.

UNIVERSITÄTS KLINIKUM FRANKFURT GOETHE-UNIVERSITÄT

a) Verwendung Ihrer Bioproben

Mit Hilfe der Bioproben soll die Unbedenklichkeit oder Wirksamkeit von IMU-838 bei Menschen mit Post COVID Syndrom klinisch untersucht werden.

Zudem können die Bioproben auch bei den notwendigen Zulassungsverfahren für das zu untersuchende Arzneimittel verwendet werden.

Sie werden zu weiteren optionalen Verwendungszwecken gesondert angesprochen, entsprechend aufgeklärt und um eine separate Einwilligung gebeten.

Ihre Proben werden Eigentum von der Goethe-Universität Frankfurt. Dessen ungeachtet bleiben Ihre Rechte, die Ihre Persönlichkeit im Zusammenhang mit dem Körpermaterial schützen, davon unberührt.

b) Lagerung

Die Aufbewahrung der Bioproben erfolgt unter Verantwortung des lokalen Studienleiters. Sie werden nach Ende der Studie vernichtet, soweit keine gesetzliche Aufbewahrungspflicht besteht oder Sie gesondert ihre Einwilligung zur weiteren Nutzung für spätere medizinische Forschungsprojekte erteilen.

Die Bioproben werden unter Verantwortung des Sponsors ausschließlich innerhalb der Europäischen Union gelagert.

c) Weitergabe/Empfänger

Die Bioproben werden im Auftrag des Sponsors an Analyselabore weitergegeben. Es gelten entsprechend die Ausführungen oben II. 1. e), insbesondere auch zur Weitergabe in andere Länder.

d) Umgang mit den Bioproben bei Widerruf/vorzeitiger Beendigung der Teilnahme

Wenn Sie Ihre Teilnahme an der klinischen Prüfung vorzeitig beenden möchten, werden Ihre Proben vernichtet, außer, diese müssen weiterhin für die Studie vorgehalten werden. Es gelten entsprechend die Ausführungen oben II. 1. f).

Einwilligungserklärung zu einer klinischen Prüfung (Studie) mit optionalem MRT

Prüfzentrum:

xy

Hauptprüfer oder einziger Prüfer: XXX

EU trial number: 2024-511628-16-00

Name und Anschrift des Sponsors: Goethe-Universität Frankfurt, vertreten durch den Präsidenten, dieser vertreten durch [redacted]

Universitätsklinikum Frankfurt
Theodor-Stern-Kai 7
60590 Frankfurt
Tel.: [redacted]
E-Mail: [redacted]

Randomisierte Studie zur Untersuchung von Behandlungsoptionen des Post-COVID Syndroms

Randomized adaptive assessment of post COVID syndrome treatments_Reducing Inflammatory Activity in Patients with post COVID Syndrome

Prüfplancode: RAPID_REVIVE

Die grauen Felder sind von dem/der Ärzt*in auszufüllen

**pheno _ _ _ _ _ _ _ _ _ _**
Codierung erfolgt nach Unterschrift

Name, Vorname des/der Patient*in	□ m □ w □ d	Geburtsdatum:
		Geburtsort:

Ich bin in einem persönlichen Gespräch durch den Prüfarzt ausführlich und verständlich über Wesen, Bedeutung, Risiken und Tragweite der klinischen Prüfung aufgeklärt worden. Ich habe darüber hinaus den Text der Patienteninformation mit seinen beiden Teilen (Teil I: Informationen zu gesundheitlichen Aspekten; Teil II: Informationen zur Verwendung der Daten und Bioproben) gelesen und verstanden. Ich hatte die Gelegenheit, mit dem Prüfarzt über die Durchführung der klinischen Prüfung zu sprechen. Alle meine Fragen wurden zufriedenstellend beantwortet.

Ich hatte ausreichend Zeit, mich zu entscheiden.

Mir ist bekannt, dass ich jederzeit und ohne Angabe von Gründen meine Einwilligung zur Teilnahme an der klinischen Prüfung zurückziehen kann (mündlich oder schriftlich), ohne dass mir daraus Nachteile entstehen.

Ergänzungen durch die aufklärende Person:

Folgende wesentliche Gesichtspunkte oder Fragen sind in dem mündlichen Aufklärungsgespräch genauer besprochen worden. Ich habe mich dabei davon überzeugt, dass der Patient alle für ihn wichtigen Fragen stellen konnte und die Aufklärung für ihn verständlich war.

Datenschutzrechtliche Einwilligung

I. Mir ist bekannt, dass bei dieser klinischen Prüfung **personenbezogene Daten**, insbesondere medizinische Befunde über mich erhoben, gespeichert und ausgewertet werden sollen. Die Verwendung meiner personenbezogenen Daten setzt vor der Teilnahme an der klinischen Prüfung folgende freiwillig abgegebene Einwilligungserklärung voraus; ohne die nachfolgende Einwilligung kann ich nicht an der klinischen Prüfung teilnehmen.

- Ich willige ein, dass im Rahmen dieser klinischen Prüfung personenbezogene Daten, insbesondere Angaben über meine Gesundheit und meine ethnische Herkunft, über mich erhoben und in Papierform sowie auf elektronischen Datenträgern gemäß den Angaben in der Informationsschrift Teil II 1. aufgezeichnet, verwendet und weitergegeben werden. Die dort genannten gesetzlichen Einschränkungen meiner Rechte sind mir bewusst.

II. Ich willige ein, dass meine **Bioproben** gemäß den Angaben der Informationsschrift Teil II 2. beschrieben aufbewahrt, verwendet und weitergegeben werden.

Das Eigentum an den Bioproben übertrage ich an den Sponsor

III. Bildgebungsverfahren des Kopfes mittels MRT

Ich wurde über die Risiken des Verfahrens und die Möglichkeit informiert, dass Zufallsbefunde bei der MRT Untersuchung erhoben werden können (gemäß den Angaben der Patienteninformation Teil I.5.b).

– Ich willige in die Teilnahme an dem Bildgebungsverfahren des Kopfes mittels MRT ein. Ich willige ein, dass im Fall eines Zufallsbefundes, dieser mit Fachärzten besprochen wird. Ich willige weiterhin ein, dass ich von [der/dem Name der behandelnden Einrichtung] wieder kontaktiert werden darf, um über medizinische Zufallsbefunde informiert zu werden. Mir ist bewusst, dass ein MRT des Kopfes nur durchgeführt werden kann, wenn ich in dieses Vorgehen eingewilligt habe.

□ Ja □ Nein

Ich willige freiwillig ein, an der oben genannten klinischen Prüfung teilzunehmen.

Zugleich willige ich in die Verarbeitung meiner personenbezogenen Daten und Bioproben wie beschrieben und von mir angegeben ein.

Ein Exemplar der Patienten-Information und -Einwilligung sowie die Versicherungsunterlagen habe ich erhalten. Ein Exemplar verbleibt im Prüfzentrum.

Die mir ausgehändigten Anlagen 1-3 gehören ebenso zu den Informationsunterlagen.

____

Name, Vorname des Patienten in Druckbuchstaben

____

Ort, Datum Unterschrift des **Patienten**

Ich habe das Aufklärungsgespräch geführt und die Einwilligung des Patienten eingeholt.

____

Name, Vorname des aufklärenden Arztes des Prüfzentrums in Druckbuchstaben

____

Ort, Datum Unterschrift des aufklärenden **Arztes des Prüfzentrums**

Anlagen

Anlage 1: Terminplan
Anlage 2: Gebrauchsanweisung Ohrsensor
Anlage 2: Gebrauchsanweisung Armbanduhr
Anlage 3: Nebenwirkungen
