# Supplementary material for: Randomized adaptive assessment of post COVID syndrome treatments (RAPID): a study protocol for a multicenter, randomized, controlled adaptive platform trial of treatment options for Post Covid Syndrome (PCS) on patients physical function including the first intervention specific appendix RAPID_REVIVE (reducing inflammatory activity in patients with PCS)
Source: Trials. 2025 Aug 19;26:297. doi: 10.1186/s13063-025-09008-0 (PMC12366011; doi:10.1186/s13063-025-09008-0)
Supplement: Supplementary file 3 — Additional file 3. [file 13063_2025_9008_MOESM3_ESM.pdf]

Patienteninformation
zur Optionalen zusätzlichen Sammlung von Bioproben und Nutzung von Daten im Rahmen der klinischen Arzneimittelprüfung:

Randomisierte Studie zur Untersuchung von Behandlungsoptionen des Post-COVID Syndroms

Randomized adaptive assessment of post COVID syndrome treatments Reducing Inflammatory Activity in Patients with post COVID Syndrome

Prüfplancode: RAPID_REVIVE

Prüfzentrum:

Platzhalter

Sehr geehrte Studieninteressentin/ sehr geehrter Studieninteressent,

Sie erwägen eine Teilnahme an der Studie ‚Randomized adaptive assessment of post COVID syndrome treatments_Reducing Inflammatory Activity in Patients with post COVID Syndrome' (nachfolgend Hauptstudie genannt).

Wir möchten Sie fragen, ob Sie darüber hinaus bereit sind, Restbiomaterial aus der Studie und eine zusätzliche Blutprobe/Gewebeprobe (in Ihrem Fall eine Blut- und eine Speichelprobe; nachfolgend Bioproben genannt) für prüfzusätzliche Untersuchungen zur Verfügung zu stellen.

Die für die Studie erhobenen Daten haben einen hohen wissenschaftlichen Wert. Auch diese würden wir gerne für weitere Forschungsprojekte nutzen.

Diese zusätzliche Probensammlung und die weitere Nutzung der Studiendaten ist freiwillig und erfolgt nur, wenn Sie dazu schriftlich Ihre Einwilligung erklären. **Auch wenn Sie ihr nicht zustimmen, können Sie dennoch an der Hauptstudie teilnehmen. Soweit Sie sich nicht beteiligen oder Ihre Zustimmung später widerrufen möchten, erwachsen Ihnen daraus keine Nachteile.**

Im Folgenden informieren wir Sie über die Ziele der Proben- und Datensammlung, die Verfahrensweisen und die Maßnahmen zum Schutz Ihrer personenbezogenen Daten, damit Sie sich auf dieser Grundlage Ihre eigene Meinung bilden und eine Entscheidung treffen können.

1. Warum werden die Proben gesammelt?

Die gesammelten Bioproben und zugehörigen Daten werden in einer sogenannten Biobank aufbewahrt und für Forschungszwecke zur Verfügung gestellt, um die Vorbeugung, Erkennung und Behandlung von Erkrankungen zu verbessern.

Untersuchungen von menschlichen Bioproben und die Analyse der daraus gewonnenen oder zu gewinnenden Daten sind zu einem wichtigen Instrument medizinischer Forschung geworden. So wissen wir heute, dass zum Beispiel genetische Eigenschaften bei der Entstehung und Behandlung von Krankheiten eine wichtige Rolle spielen und auch die Wirkung von Medikamenten beeinflussen können.

Das Ziel dieser Forschung ist nicht, bei Ihnen oder anderen einzelnen Personen eine Diagnose zu erstellen. Vielmehr sollen bei der vergleichenden Untersuchung von größeren Personengruppen biomedizinische Zusammenhänge ermittelt werden.

Die Nutzung der Bioproben und Daten werden durch die organisatorisch für den regulierten Herausgabeprozess verantwortlichen Partner des Netzwerk Universitätsmedizin (NUM) verantwortet. Das NUM ist ein bundesweites Netzwerk aller Universitätsklinika Deutschlands und ist als nationale Koordinierungsstelle aktuell bei der Charité Universitätsmedizin Berlin angesiedelt.

Das NUM wurde im Jahr 2020 als Teil des Krisenmanagements gegen die COVID-19-Pandemie gegründet, um die klinische COVID-19-Forschung der gesamten Universitätsmedizin zu koordinieren. Seitdem wurden die organisatorischen und technischen Rahmenbedingungen stetig weiterentwickelt und können für weitere Forschungsprojekte genutzt werden.

2. Um welche Art von Bioproben und Daten handelt es sich?

Bei der Bioprobe handelt es sich um Körperflüssigkeiten, die ohnehin im Rahmen der Hauptstudie entnommen, dafür jedoch nicht mehr benötigt werden und daher ansonsten vernichtet würden.

Hinzu kommen ca. 10 ml Blutproben und eine Speichelprobe, die zusätzlich bei jeder Visite gewonnen werden.

Bei den Daten handelt es sich um die medizinischen Daten, die im Rahmen der Hauptstudie erhoben werden. Darüber hinaus können weitere Daten bei der Untersuchung der Bioproben gewonnen werden (siehe Punkt 3.).

Außerdem besteht die Möglichkeit, einer Verknüpfung von Daten aus Ihrer Krankenversicherung zuzustimmen (siehe Punkt 7 b.)

3. Wie werden die Bioproben und Daten verwendet?

Wir fragen Sie nach einer sehr breit gefassten Erlaubnis zur Verwendung Ihrer Bioproben und Daten. Diese werden für medizinische Forschung bereitgestellt, die die Vorbeugung, Erkennung und Behandlung von Erkrankungen verbessern soll. Sie sollen im Sinne eines möglichst großen Nutzens für die Allgemeinheit für viele verschiedene medizinische Forschungszwecke verwendet werden.
Diese können sich sowohl auf bestimmte Krankheitsgebiete (z.B. Krebsleiden, Herz-Kreislauf-Erkrankungen, Erkrankungen des Gehirns) als auch auf heute zum Teil noch unbekannte

Krankheiten und genetische Zusammenhänge beziehen. Weil sich in der Forschung immer wieder neue Fragen ergeben, kann es sein, dass Ihre Proben und Daten auch für medizinische Forschungsvorhaben verwendet werden, die man heute noch nicht absehen kann. Ihre Bioproben und Daten werden nicht für Forschungsvorhaben verwendet, die von der Ethik-Kommission, die das Vorhaben bewertet, als unethisch erachtet werden (siehe Punkt 7e).

Möglicherweise werden an Ihren Bioproben auch genetische Untersuchungen durchgeführt, und zwar unter Umständen auch eine Untersuchung Ihrer gesamten Erbsubstanz (Genom).

Aus logistischen Gründen ist es nicht möglich, individuelle Eingrenzungen (z.B. Ausschluss bestimmter Forschung, Ausschluss der Weitergabe der Materialien an Dritte) vorzunehmen. Wenn Sie mit der beschriebenen Art und Dauer der Nutzung nicht in vollem Umfang einverstanden sind, sollten Sie Ihre Einwilligung nicht erteilen.

Um für die Gesellschaft einen möglichst großen Nutzen aus öffentlich finanzierten Ressourcen zu erbringen, sollen die Bioproben und Daten für unbestimmte Zeit aufbewahrt und für die medizinische Forschung bereitgestellt werden. Ein Zeitpunkt, zu welchem Daten/Proben nicht mehr forschungsrelevant sind, ist vorab nicht bestimmbar. Deshalb findet eine regelmäßige Überprüfung statt, ob Daten und Proben noch forschungsrelevant sind. Wenn das nicht der Fall ist, werden diese vernichtet.

4. Welche Risiken sind mit Ihrer Spende verbunden?

a) Gesundheitliche Risiken

Bei Ihnen ist im Rahmen der Hauptstudie ohnehin eine Blutentnahme geplant. Dabei möchten wir 10 ml Blut zusätzlich abnehmen (das entspricht etwa einem Esslöffel). Diese Entnahme ist für Sie mit keinem zusätzlichen gesundheitlichen Risiko verbunden.

b) Weitere Risiken

Bei jeder Erhebung, Speicherung und Übermittlung von Daten zu Ihren Bioproben im Rahmen von Forschungsprojekten bestehen Vertraulichkeitsrisiken (z.B. die Möglichkeit, Sie zu identifizieren), insbesondere im Hinblick auf die Information zu Ihrer Erbsubstanz. Diese Risiken lassen sich nicht völlig ausschließen und steigen, je mehr Daten miteinander verknüpft werden können, insbesondere auch dann, wenn Sie selbst (z.B. zur Ahnenforschung) genetische Daten im Internet veröffentlichen. Unter Punkt 7 „Was geschieht mit Ihren Bioproben und Daten?“ erläutern wir Ihnen genauer, wie Ihre Privatsphäre geschützt wird.

5. Welcher Nutzen ergibt sich für Sie persönlich?

Persönlich können Sie für Ihre Gesundheit keinen unmittelbaren Vorteil oder Nutzen aus der Spende Ihrer Proben und Daten erwarten. Deren Auswertung dient ausschließlich Forschungszwecken und nicht dazu, Rückschlüsse auf Ihre Gesundheit zu ziehen.

Es ist jedoch im Einzelfall möglich, dass ein Forscher zu der Einschätzung gelangt, dass ein Auswertungsergebnis für Ihre Gesundheit von erheblicher Bedeutung sein könnte. Das ist insbesondere der Fall, wenn sich daraus ein dringender Verdacht auf eine schwerwiegende, bisher möglicherweise nicht erkannte Krankheit ergibt, die behandelt oder deren Ausbruch verhindert werden könnte. In einem solchen Fall kann eine Rückmeldung an Sie erfolgen (siehe Punkt 10).

Bitte kreuzen Sie in der Einwilligungserklärung an, ob Sie in einem solchen Fall eine Rückmeldung erhalten möchten (siehe Punkt 10). **Sie können Ihre Entscheidung für oder gegen eine Rückmeldungsmöglichkeit jederzeit durch Mitteilung an Ihr Prüfzentrum oder nach Ende der klinischen Studie an die unabhängige Treuhandstelle ändern. Beachten Sie, dass Sie Gesundheitsinformationen, die Sie durch eine solche Rückmeldung erhalten, unter Umständen bei anderen Stellen (z.B. vor Abschluss einer Kranken- oder Lebensversicherung) offenbaren müssen und dadurch Nachteile erleiden können.**

Da auch Untersuchungen Ihrer Erbsubstanz möglich/vorgesehen sind, kann sich der vorstehende Text auch auf Ihre genetischen Veranlagungen, z.B. für bestimmte Erkrankungen, beziehen. Informationen zu Ihrer Erbsubstanz können auch Auswirkungen auf Ihre Familienangehörigen und die Familienplanung haben.

6. Welcher Nutzen ergibt sich für die Allgemeinheit?

Medizinisch-wissenschaftliche Forschungsvorhaben zielen auf eine Verbesserung unseres Verständnisses der Krankheitsentstehung und der Diagnosestellung und auf dieser Basis auf die Entwicklung von verbesserten Behandlungsansätzen, insbesondere Arzneimitteln. Informationen über die Aktivitäten des NUM und Projekten, die mit Biomaterialien und Daten des NUM forschen, finden Sie unter https://www.netzwerk-universitaetsmedizin.de

7. Was geschieht mit Ihren Bioproben und Daten und wie werden sie geschützt?

a) Kodierung Ihrer Bioproben und Daten

Alle Daten, die Ihre Person unmittelbar identifizieren (Name, Geburtsdatum, Anschrift etc.), werden unverzüglich nach Gewinnung der Bioproben durch einen Code ersetzt (pseudonymisiert). Erst in dieser Form werden die Bioproben und Daten für Forschungszwecke verwendet. Spätestens bei der durch die zuständigen Partner des NUM vermittelten Herausgabe an Dritte müssen die Proben und Daten nochmals kodiert werden (doppelte Pseudonymisierung).

Die Sie unmittelbar identifizierenden Daten werden getrennt von den Bioproben und medizinischen Daten nur im Prüfzentrum und der Unabhängigen Treuhandstelle an der Universitätsmedizin Greifswald gespeichert. Die Proben und Daten können deshalb nicht ohne Mitwirkung des Prüfzentrums oder der Treuhandstelle Ihrer Person zugeordnet werden. Eine solche Zuordnung erfolgt nur, um zusätzliche Daten aus Ihren Krankenunterlagen zu ergänzen oder erneut mit Ihnen in Kontakt zu treten, falls Sie der Kontaktaufnahme zugestimmt haben

(siehe Punkt 10). **Eine Weitergabe der Ihre personenidentifizierenden Daten an den Sponsor, andere Forscher oder sonstige unberechtigte Dritte, etwa Versicherungsunternehmen oder Arbeitgeber, erfolgt nicht.**

b) Verknüpfung von Daten aus Ihrer Krankenversicherung

Übertragung und wissenschaftliche Nutzung von Krankenkassendaten

Für viele wissenschaftliche Fragestellungen reichen Daten aus der Hauptstudie häufig nicht aus. Um ein umfassenderes Bild von Ihrem Gesundheitszustand zu erhalten, würden wir z.B. gerne auch Ihre Patientendaten aus der ambulanten Versorgung nutzen. Über diese Informationen verfügt Ihre Krankenkasse. Wir bitten Sie darum, dass wir von Ihnen auch Daten, z.B. über vorangegangene und nachfolgende Arztkontakte bei ambulanten Haus- und Fachärzten sowie ggf. von weiteren Krankenhausaufenthalten und Arzneimittel-Verordnungen anfordern und wissenschaftlich nutzen dürfen. In der Einwilligungserklärung können Sie uns dazu ermächtigen, die entsprechenden Daten bei Ihrer Krankenkasse anzufordern. Die Krankenkassen erhalten von uns aber keinerlei Forschungsergebnisse, die Ihnen persönlich zugeordnet werden könnten. Damit wird Ihnen auch kein Nachteil durch die Nutzung Ihrer Krankenkassen-Daten entstehen.

c) Die Lagerung und Weitergabe von Bioproben und Daten

Die kodierten Bioproben und medizinischen Daten werden entweder an Ihrem lokalen Prüfzentrum oder an der zentralen Biobank eines Partners des NUM aufbewahrt und zu Forschungszwecken verwendet. Dabei werden die Daten unter Umständen auch mit medizinischen Daten in anderen Datenbanken verknüpft, sofern die gesetzlichen Voraussetzungen hierfür erfüllt sind.

Die Bioproben und Daten können für genauer bestimmte medizinische Forschungszwecke nach zuvor festgelegten Regeln auch an Dritte (z.B. Universitäten, Forschungsinstitute und forschende Unternehmen) weitergegeben werden. Diese Bioproben und Daten dürfen nur für den vorbestimmten Forschungszweck verwendet und vom Empfänger nicht zu anderen Zwecken weitergegeben werden. Nicht verbrauchtes Material wird an das NUM zurückgegeben oder vernichtet.

d) Lagerung und Weitergabe ins Ausland

Innerhalb der Europäischen Union und des Europäischen Wirtschaftsraumes können Ihre Daten und Bioproben auf Basis gemeinsamer Datenschutzgesetzgebung weitergegeben werden.

Für Länder, für die die Europäische Kommission ein angemessenes gesetzliches Datenschutzniveau festgestellt hat (Angemessenheitsbeschluss), gilt dies ebenso.

Wenn Sie dem zustimmen, können Ihre Bioproben und Daten auch in Länder außerhalb der Europäischen Union weitergegeben werden, die nicht diese Voraussetzungen erfüllen.

Diese Länder haben **möglicherweise ein niedrigeres Datenschutzniveau** als die EU. Das NUM sichert zu, soweit rechtlich möglich, das EU-Datenschutz-Niveau einzuhalten und seine

Forschungspartner vertraglich ebenfalls dazu zu verpflichten. Dennoch besteht das Risiko, dass staatliche oder private Stellen auf Ihre Daten zugreifen, obwohl dies nach dem europäischen Datenschutzrecht nicht zulässig wäre. Zudem kann es sein, dass Ihnen dort weniger oder schlechter durchsetzbare Betroffenenrechte zustehen und es keine unabhängige Aufsichtsbehörde gibt, die Sie bei der Wahrnehmung Ihrer Rechte unterstützen könnte. **Eine Weitergabe Ihrer Bioproben und Daten kann in diese Länder nur erfolgen, wenn Sie dem ausdrücklich zugestimmt haben. Dazu können Sie in der Einwilligungserklärung das entsprechende Kästchen ankreuzen.**

e) Bewertung durch die Ethik-Kommission

Voraussetzung für die Verwendung der Bioproben und Daten für ein konkretes medizinisches Forschungsprojekt ist grundsätzlich, dass das Forschungsvorhaben durch eine unabhängige Ethik-Kommission zustimmend bewertet wurde.

f) Veröffentlichungen

Wissenschaftliche Veröffentlichungen von Ergebnissen erfolgen ausschließlich in einer Form, die keine Rückschlüsse auf Ihre Person zulässt.

Das gilt insbesondere auch für genetische Informationen. Möglich ist allerdings eine Aufnahme genetischer Informationen in besonders geschützte wissenschaftliche Datenbanken, die für die Allgemeinheit nicht zugänglich sind.

8. Erlangen Sie einen finanziellen Vorteil aus der Nutzung Ihrer Bioproben und Daten?

Mit der Überlassung der Bioproben an Ihr Prüfzentrum geht das Eigentum an den Bioproben zu 1/3 auf Ihr Prüfzentrum und zu 2/3 auf das NUM über. Ferner ermächtigen Sie das NUM, Ihre Daten zu nutzen. Für die Überlassung Ihrer Bioproben und Daten erhalten Sie kein Entgelt. Sollte aus der Forschung ein kommerzieller Nutzen erzielt werden, werden Sie daran nicht beteiligt.

Das NUM verwendet Ihre Bioproben und Daten ausschließlich für wissenschaftliche Zwecke. Die Proben und Daten werden nicht verkauft.

Das NUM kann jedoch für die Weitergabe der Bioproben und Daten an Dritte eine angemessene Aufwandsentschädigung erheben.

9. Gibt es eine Versicherung?

Die zusätzliche Entnahme von Bioproben ist über die Versicherung der Hauptstudie miterfasst. Nähere Erläuterungen zu der Versicherung haben Sie im Rahmen der Hauptstudie erhalten.

10. Erfolgt eine erneute Kontaktaufnahme mit Ihnen?

Zur Erhebung von weiteren Verlaufsdaten kann es sinnvoll werden, zu einem späteren Zeitpunkt erneut Kontakt mit Ihnen aufzunehmen, um ergänzende Informationen und/oder Bioproben von Ihnen zu erbitten. Zudem kann die erneute Kontaktaufnahme genutzt werden um zum Beispiel Ihnen/Ihrem behandelnden Arzt/Studienarzt/Hausarzt eine Rückmeldung über für Sie gesundheitlich relevante Ergebnisse zu geben (siehe oben Punkt 5).

Kreuzen Sie in der Einwilligungserklärung bitte an, ob Sie eine erneute Kontaktaufnahme in diesen Fällen wünschen oder nicht.

11. Was beinhaltet Ihr Widerrufsrecht?

Sie können Ihre Einwilligung in die Aufbewahrung und Verwendung Ihrer Proben jederzeit ohne Angabe von Gründen und ohne nachteilige Folgen für Sie widerrufen. Die Rechtmäßigkeit der bis zum Widerruf erfolgten Nutzung der Proben und Daten bleibt davon jedoch unberührt.

Im Falle des Widerrufs werden die Bioproben vernichtet und die Daten für weitergehende Nutzung gesperrt. Eine Datenlöschung kann nur erfolgen, wenn diese nicht mehr für Nachweiszwecke benötigt werden und dies mit zumutbarem technischem Aufwand möglich ist. Zudem können Daten aus bereits durchgeführten Analysen nicht mehr entfernt werden.

Wenn Daten aus der Probensammlung auch in die Hauptstudie eingeführt wurden, besteht für Sie ein Löschungsanspruch nur im Rahmen der Vorgaben des Arzneimittelgesetzes (siehe Einwilligungserklärung zur Hauptstudie).

Wenden Sie sich für einen Widerruf bitte an:

Unabhängige Treuhandstelle der Universitätsmedizin Greifswald
Ellernholzstr. 1-2
17475 Greifswald
https://www.ths-greifswald.de/kontakt/

12. Welche weiteren Datenschutzrechte haben Sie?

Rechtsgrundlage für die Datenverarbeitung ist Ihre Einwilligung gemäß Art. 6 Abs. 1 Buchst. a und Art. 9 Abs. 2 Buchst. a der Datenschutz-Grundverordnung.

Verantwortlicher im Sinne der Datenschutz-Grundverordnung ist:

Sponsor:
Goethe-Universität Frankfurt, vertreten durch den Präsidenten, dieser vertreten durch Prof. Dr. [redacted]

Universitätsklinikum Frankfurt
Theodor-Stern-Kai 7

60590 Frankfurt
Tel.: [redacted]

[redacted]

Prüfzentrum (jeweiliges):

Platzhalter

Verantwortlich für die Datenhaltung: Personendaten und Identifizierungsschlüssel:
Unabhängige Treuhandstelle Greifswald
An der Universitätsmedizin Greifswald
Institut für Community Medicine, Abt. VC
Ellernholzstr. 1-2, 17475 Greifswald

Medizinische Daten:
Datenhaltung und Transferstelle
Institut für Medizinische Informatik
Universitätsmedizin Göttingen der Georg-August-Universität
Robert-Koch-Str. 40, 37075 Göttingen

Bilddatenmanagement-System (DIMA):
Koordinator des DIMA
Charité – Universitätsmedizin Berlin
Klinik für Pädiatrie mit Schwerpunkt Kardiologie
Augustenburger Platz 1, 13353 Berlin

Laborinformationssystem:
Systemadministration Laborinformationssystem:
Institut für Klinische Chemie und Laboratoriumsmedizin
Universitätsmedizin Greifswald
Ferdinand-Sauerbruch-Straße, 17475 Greifswald .

Sie können vom Prüfzentrum und den oben genannten datenhaltenden Stellen im Rahmen der gesetzlichen Vorgaben Auskunft über Ihre dort gespeicherten Daten verlangen. Ebenso können Sie eine Berichtigung falscher Daten, eine Übertragung der von Ihnen zur Verfügung gestellten Daten sowie eine Löschung der Daten oder Einschränkung ihrer Verarbeitung verlangen. Für die Ausübung dieser Rechte können Sie sich an das Prüfzentrum und die zuständige Stelle des NUM, vertreten durch die Koordinierungsstelle an der Charité, wenden. Bitte wenden Sie sich im Regelfall an das Prüfzentrum, denn das Prüfzentrum kann zuordnen, welche Daten zu Ihnen gehören. Nach Beendigung der klinischen Prüfung steht Ihnen außerdem die unabhängige Treuhandstelle Greifswald als Kontaktstelle zur Verfügung.

Bei Anliegen zur Datenverarbeitung und zur Einhaltung des Datenschutzes können Sie sich auch an folgende Datenschutzbeauftragte wenden:

Datenschutzbeauftragter Charité (NUM Koordinierungsstelle):

Behördliche Datenschutzbeauftragte der Charité – Universitätsmedizin Berlin
Charitéplatz 1
10117 Berlin
E-Mail: datenschutz@charite.de

Sie haben außerdem ein Beschwerderecht bei jeder Datenschutzaufsichtsbehörde. Eine Liste der Aufsichtsbehörden in Deutschland finden Sie unter:

https://www.bfdi.bund.de/DE/Infothek/Anschriften_Links/anschriften_links-node.html.

13. Wo können Sie weitere Informationen erhalten?

Sollte Ihnen etwas unklar sein, fragen Sie bitte Ihren Prüfarzt, bevor Sie Ihre Einwilligung erteilen. Sie können sich wegen Rückfragen auch zu einem späteren Zeitpunkt an Ihre Prüfstelle wenden solange Sie an der Studie teilnehmen. Bezüglich Ihrer Betroffenenrechte steht außerdem die Treuhandstelle Greifswald langfristig als Ansprechpartner zur Verfügung.

Weitere Informationen zu Forschungsprojekten, die Daten und/oder Bioproben des NUM erhalten haben, finden Sie unter https://www.netzwerk-universitaetsmedizin.de/

Einwilligungserklärung

zur Optionalen zusätzlichen Sammlung von Bioproben und Nutzung von Daten im Rahmen der klinischen Arzneimittelprüfung:

Randomisierte Studie zur Untersuchung von Behandlungsoptionen des Post-COVID Syndroms

Randomized adaptive assessment of post COVID syndrome treatments_Reducing Inflammatory Activity in Patients with post COVID Syndrome

Prüfplancode: RAPID_REVIVE

Bitte lesen Sie die folgende Einwilligungserklärung aufmerksam durch, kreuzen Sie Zutreffendes an und unterschreiben Sie anschließend am Ende dieser Einwilligungserklärung, sofern Sie einverstanden sind.

Die grauen Felder sind von dem/der Ärzt*in auszufüllen

pheno _ |_|_|_|_|_|_|_|_|_|

Codierung erfolgt nach Unterschrift

Name, Vorname des/der Patient*in	☐ m ☐ w ☐ d	Geburtsdatum:
		Geburtsort:

Ich habe die Informationsschrift zur zusätzlichen Proben- und Datensammlung gelesen und hatte die Gelegenheit, Fragen zu stellen. Ich weiß, dass meine Teilnahme freiwillig und unabhängig von der Hauptstudie ist. Ich kann meine Einwilligung jederzeit ohne Angabe von Gründen widerrufen, ohne dass mir daraus irgendwelche Nachteile entstehen.

I. Ich willige ein, dass meine Bioproben und Daten, wie in der Informationsschrift beschrieben, an mein Prüfzentrum gegeben und für die in der Informationsschrift genannten medizinischen Forschungszwecke verwendet werden. Die Nutzung für weitere Forschungsprojekte wird durch die für den regulierten Herausgabeprozess verantwortlichen Partner des NUM reguliert und vermittelt.

II. Insbesondere willige ich ein, dass, wie in der Informationsschrift beschrieben,

- die von meinem Prüfzentrum erhobenen personenbezogene Daten, insbesondere Angaben über meine Gesundheit, für weitere medizinische Forschungszwecke verwendet werden;

- die Bioproben pseudonymisiert an meinem Prüfzentrum oder an der zentralen Biobank eines Partners des Netzwerk Universitätsmedizin (NUM) aufbewahrt und verwendet werden. Das Eigentum an den Bioproben übertrage ich an mein Prüfzentrum und an das NUM;

- durch die Untersuchung dieser Bioproben Daten über meine Gesundheit erhoben werden und durch die Partner des NUM in pseudonymisierter Form gespeichert werden. Diese Daten werden ggf. mit den ebenfalls pseudonymisierten Daten aus der Hauptstudie verknüpft und für die in der Informationsschrift genannten medizinischen Forschungszwecke verwendet.

- die Bioproben mit den vorgenannten Daten pseudonymisiert an Universitäten, Forschungsinstitute und forschende Unternehmen zu Zwecken medizinischer Forschung weitergegeben werden dürfen.

Dies schließt auch die Lagerung bzw. Speicherung und Weitergabe außerhalb der EU ein. Dies ist generell zulässig, wenn ein Angemessenheitsbeschluss der Europäischen Kommission vorliegt.

III. Ich willige außerdem in die Lagerung bzw. Speicherung und Weitergabe meiner Bioproben und Daten in Länder außerhalb der EU auch in den Fällen ein, in denen kein Angemessenheitsbeschluss der Europäischen Kommission vorliegt. Über die möglichen Risiken einer solchen Weitergabe bin ich aufgeklärt worden (Punkt 7d in der Information).

☐ ja ☐ nein

IV. Ich willige ein, dass

- meine für die Studie erhobenen Daten mit medizinischen Daten aus anderen Datenbanken verknüpft werden

  ☐ ja ☐ nein

- meine Krankenkassendaten zur wissenschaftlichen Nutzung übertragen werden.

  Hiermit ermächtige ich meine Krankenkasse auf Anforderung durch das NUM, Daten über von mir in Anspruch genommene ärztliche Leistungen in der ambulanten Versorgung und bei stationären Aufenthalten, über verordnete Heil- und Hilfsmittel sowie Arzneimittel und Angaben zum Bereich Pflege an die dafür zuständigen Partner des NUM, so wie in der Patienteninformation beschrieben, zu übermitteln, und zwar:

  - Einmalig rückwirkend für die Daten der vergangenen 5 Kalenderjahre. Mit der dafür nötigen Übermittlung meiner Krankenversicherungs-Nummer an das NUM bin ich einverstanden

    ☐ ja ☐ nein

  - Für Daten ab dem Datum meiner Unterschrift über einen Zeitraum von 5 Jahren. Mit der dafür nötigen Übermittlung meiner Krankenversicherungs-Nr. an das NUM bin ich einverstanden

    ☐ ja ☐ nein

V. Ich willige ein, dass ich eventuell zu einem späteren Zeitpunkt erneut kontaktiert werde

- zum Zweck der Gewinnung weiterer Informationen/Bioproben

  ☐ ja ☐ nein

- zum Zweck der Rückmeldung für mich wichtiger gesundheitsrelevanter Ergebnisse

  ☐ ja ☐ nein

Diese Rückmeldung soll erfolgen über die Einrichtung, in der meine Bioproben/Daten gewonnen wurden oder über folgenden Arzt (falls gewünscht, bitte angeben):

Name und Anschrift des Arztes: ____

Eine Kopie der Patienten-/Probandeninformation und Einwilligungserklärung habe ich erhalten. Das Original verbleibt im Prüfzentrum.

____
Name, Vorname des Patienten/Probanden in Druckbuchstaben

____
Ort, Datum (vom Patient/Proband einzutragen) Unterschrift des Patienten/Probanden

Ich habe das Aufklärungsgespräch geführt und die Einwilligung des Patienten/Probanden eingeholt.

____
Name, Vorname der aufklärenden Person in Druckbuchstaben

____
Ort, Datum Unterschrift der aufklärenden Person
